# *manymome*: An R package for computing the indirect effects, conditional effects, and conditional indirect effects, standardized or unstandardized, and their bootstrap confidence intervals, in many (though not all) models

**DOI:** 10.3758/s13428-023-02224-z

**Published:** 2023-10-05

**Authors:** Shu Fai Cheung, Sing-Hang Cheung

**Affiliations:** 1 Department of Psychology, Faculty of Social Sciences, University of Macau, Avenida da Universidade, Taipa, Macao SAR, China; 2 Department of Psychology, The University of Hong Kong, Hong Kong SAR, China

**Keywords:** Mediation, Moderation, Conditional indirect effect, Structural equation modeling, Regression, Missing data, Bootstrapping

## Abstract

Mediation, moderation, and moderated mediation are common in behavioral research models. Several tools are available for estimating indirect effects, conditional effects, and conditional indirect effects and forming their confidence intervals. However, there are no simple-to-use tools that can appropriately form the bootstrapping confidence interval for standardized conditional indirect effects. Moreover, some tools are restricted to a limited type of models. We developed an R package, *manymome*, which can be used to estimate and form confidence intervals for indirect effects, conditional effects, and conditional indirect effects, standardized or not, using a two-step approach: model parameters are estimated either by structural equation modeling using *lavaan* or by a set of linear regression models using *lm*, and then the coefficients are used to compute the requested effects and form confidence intervals. It can be used when there are missing data if the model is fitted by structural equation modeling. There are only a few limitations on some aspects of a model, and no inherent limitations on the number of predictors, the number of independent variables, or the number of moderators and mediators. The goal is to have a tool that allows researchers to focus on model fitting first and worry about estimating the effects later. The use of the model is illustrated using a few numerical examples, and the limitations of the package are discussed.

It is well known that *mediation effect*, the *indirect effect* from one variable to another variable through other variables (Baron & Kenny, 1986), is popular now in behavioral research. A more complicated effect, *moderated mediation*, is a mediation effect that is moderated; that is, the indirect effect depends on the values of one or more variables (Preacher et al., 2007). The indirect effect conditional on a particular set of value(s) of the moderator(s) is called the *conditional indirect effect* (Preacher et al., 2007). This effect has been gaining popularity in behavioral research because it can enrich our understanding of an indirect effect. There are many tools available for estimating indirect effects and conditional indirect effects. For example, PROCESS (Hayes, 2022), which supports SAS, SPSS, and R, can analyze a large variety of models with mediation, moderation, and moderated mediation. However, it only supports regression models estimated by ordinary least squares (OLS) or logistic regression. The package *mediation* (Tingley et al., 2014) supports more types of models, such as multiple regression, generalized linear models, and survival regression models. Most structural equation modeling (SEM) programs also support the estimation and testing of indirect effects, although for conditional indirect effects, additional programming is usually needed. To complement existing tools in terms of models supported and the ease of use, we developed *manymome*, which allows users to fit a wide variety of models (hence *many* in the name) using either SEM or multiple regression, and then estimate and test indirect effects and conditional indirect effects in paths in the fitted model, without the need to define in advance any user parameters in the SEM models or specify in advance the paths of interest in fitting the regression models. It also yields correct bootstrap confidence intervals (CI) for standardized indirect and conditional indirect effects, the latter not easy to form and sometimes incorrectly formed using existing tools.

In this manuscript, we first review the computation of indirect effects and conditional indirect effects using the estimates of path coefficients. We then present the two-step workflow which is adopted by *manymome*. Several numerical examples are used to illustrate how to use *manymome* to estimate indirect and conditional indirect effects and form their bootstrap confidence intervals (CIs). Lastly, we compare *manymome* with some existing tools to highlight its strengths and limitations so that researchers can select the tools suitable for their data and models.

## Estimating indirect effects and conditional indirect effects

**Fig. 1:**
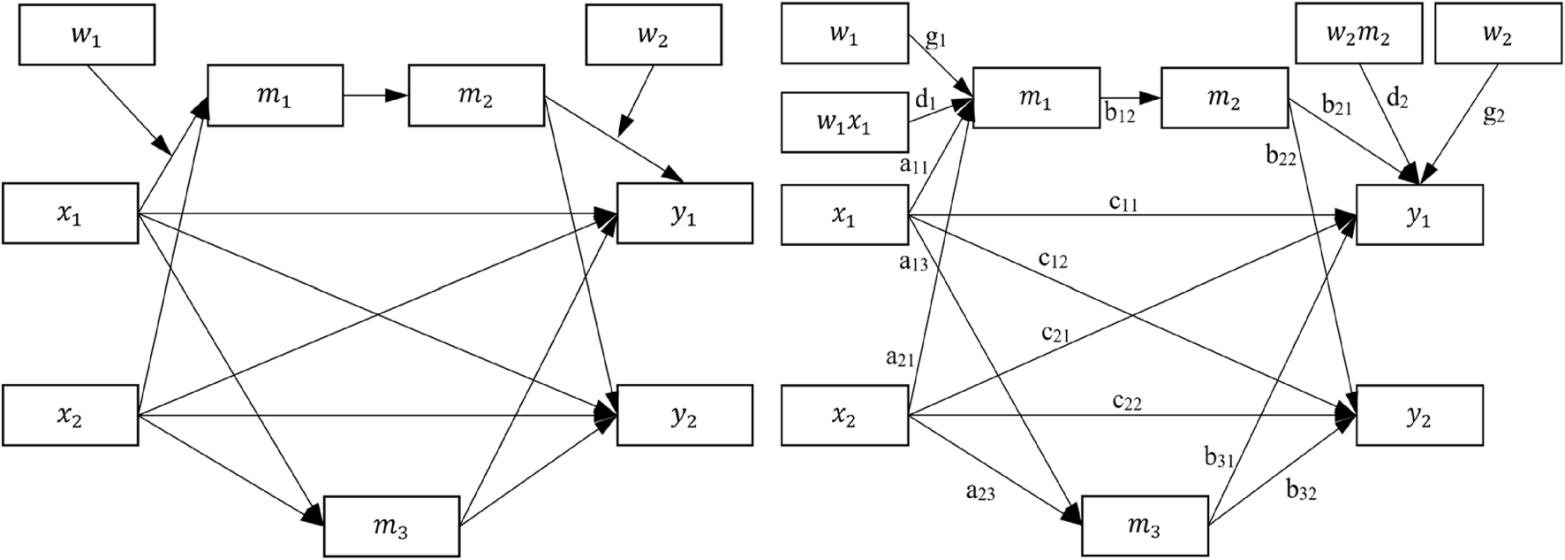
Model 1: The conceptual model (left) and the working model (right) (variances, covariances, and error terms omitted for readability)

We first briefly review how the indirect effects and conditional indirect effects in a path model are estimated, which is essential for understanding the flexibility offered by the two-step workflow adopted by *manymome* and other tools. We use the model in Fig. 1 for illustration. It has two predictors, *x*_1_ and *x*_2_, two outcome variables, *y*_1_ and *y*_2_, and three mediators. The conceptual model illustrates the role of *w*_1_ and *w*_2_ as moderators, and the working model is the model actually tested, with moderation effects modeled by the inclusion of the product terms *w*_1_*x*_1_ and *w*_2_*m*_2_. This model, though arbitrary and complicated, is designed to reflect the complexity in models in published research (e.g., Chamorro-Premuzic & Furnham, 2008, tested a model with two predictors, three mediators, and two paths; Scott & Woods, 2018, tested a model with four mediators and two paths). It includes features common in applied research: mediation paths not moderated (e.g., *x*_2_ → *m*_3_ → *y*_2_, *x*_2_ → *m*_1_ → *m*_2_ → *y*_2_), moderation (e.g., *x*_1_ → *m*_1_ moderated by *w*_1_, *m*_2_ → *y*_1_ moderated by *w*_2_), and moderated mediation (*x*_1_ → *m*_1_ → *m*_2_ → *y*_1_, with two component paths moderated). We include two predictors and two outcome variables (*y*_1_ and *y*_2_) to illustrate the advantage of using SEM to analyze a multivariate model as a whole, although we will also later discuss cases in which regression-based analysis is appropriate and sufficient. Lastly, it is common that control variables, if any, are omitted from the diagram for readability. We follow this practice in Fig. 1, although two control variables, *c*_1_ and *c*_2_, are also included in the sample dataset and numerical examples to be introduced later, to simulate real research scenarios in which control variables are common.

### Indirect effects

The indirect effect is simply the product of the path coefficients along a path. For example, in Model 1, the indirect effect in *x*_2_ → *m*_3_ → *y*_2_ is *a*_23_*b*_32_. The indirect effect in *x*_1_ → *m*_3_ → *y*_2_ is *a*_13_*b*_32_. Although *m*_1_ is usually called a mediator, if theoretically relevant, we can also estimate the indirect effect in *m*_1_ → *m*_2_ → *y*_2_, which is *b*_12_*b*_22_. The indirect effects through any other path that does not involve a moderator can be estimated similarly, regardless of where the path starts and ends.

For the *standardized indirect effect* of a path, the recommended standardized effect size measure for mediation effects (Miočević et al., 2018), regardless of the number of mediators along it, it is simply the indirect effect multiplied by *SD*_*x*_/*SD*_*y*_ (Hayes, 2022)^1^, where *SD*_*x*_ and *SD*_*y*_ are the standard deviation (*SD*) of the variable at the start of the path and the *SD* of the variable at the end of the path, respectively.^2^ For example, the standardized indirect effect in *x*_2_ → *m*_3_ → *y*_2_ is ${a}_{23}{b}_{32}\left(S{D}_{x_2}/S{D}_{y_2}\right)$. If one of the variables has a meaningful unit, a *partially standardized indirect effect* can be computed by omitting the *SD* of this variable (Hayes, 2022). For example, if *y*_2_ is monthly salary and only *x*_2_ needs to be standardized, the partially standardized indirect effect in *x*_2_ → *m*_3_ → *y*_2_ is ${a}_{23}{b}_{32}\left(S{D}_{x_2}\right)$.

Computing the point estimates of an indirect effect is simple, but testing the effect and forming an interval estimate are not simple, because the sampling distribution of the estimate, being the product of two or more estimates, is nonnormal and asymmetric (Craig, 1936). Methods that form symmetric CIs, such as the method by Sobel (1982) and delta method in SEM, were found to have suboptimal performance in some conditions, such as giving confidence intervals that tended to be too narrow (e.g., Cheung, 2009). One of the commonly used methods is nonparametric bootstrapping (Efron & Tibshirani, 1993), described below. This method has been shown to perform satisfactorily in a wide range of situations for both unstandardized and standardized indirect effects (e.g., Cheung, 2009; Falk, 2018; Pesigan & Cheung, 2020).

To form a (1 − *α*)100% percentile nonparametric bootstrap CI to estimate and test an indirect effect, *α* the level of significance for the test and (1 − *α*) the level of confidence of the CI, *R* bootstrap samples are first drawn (each sample is a random draw of *n* cases with replacement from the source sample, *n* being the sample size). In each bootstrap sample, all the component paths are estimated and then the indirect effect is computed. Lastly, the corresponding percentiles of these *R* estimates are used to form the CI. For example, the 95th percentile nonparametric bootstrap CI of the indirect effect *x*_2_ → *m*_3_ → *y*_2_ is formed by drawing *R* bootstrap samples, computing the estimates of *a*_23_ and *b*_32_ and then *a*_23_*b*_32_ in each bootstrap sample, and using the 2.5th and 97.5th percentiles of these *R* bootstrap estimates of *a*_23_*b*_32_ to form the CI. For the standardized indirect effect, it is slightly more complicated. As shown by Cheung (2009), the *SD*s also need to be recomputed in each bootstrap sample. Therefore, in each bootstrap sample, the *SD*s of *x*_2_ and *y*_2_ are also computed, and the percentiles of the ${a}_{23}{b}_{32}\left(S{D}_{x_2}/S{D}_{y_2}\right)$ in the *R* bootstrap samples are used to form the CI.

### Conditional indirect effects

When one or more component paths on a path involve one or more moderators, the indirect effect is moderated. The indirect effect computed as shown above is no longer meaningful unless the levels at which the moderators are zero are meaningful. Instead of estimating the indirect effect, it is necessary to determine the indirect effect for different levels of the moderators. The indirect effect for these levels of moderators is called the conditional indirect effect (Preacher et al., 2007).

A conditional indirect effect is also estimated by the product of the path coefficients along a path, although one or more path coefficients are computed conditional on the levels of the moderators. For example, the path *x*_1_ → *m*_1_ → *m*_2_ → *y*_1_ involves three component paths: *x*_1_ → *m*_1_, *m*_1_ → *m*_2_, and *m*_2_ → *y*_2_. However, the indirect effect cannot be computed by *a*_11_*b*_12_*b*_21_ because *x*_1_ → *m*_1_ is moderated by *w*_1_ and *m*_2_ → *y*_1_ is moderated by *w*_2_. That is, the effect from *x*_1_ to *m*_1_ is (*a*_11_ + *d*_1_*w*_1_), where *d*_1_ is the coefficient of the product term *w*_1_*x*_1_, and the effect from *m*_2_ to *y*_1_ is (*b*_21_ + *d*_2_*w*_2_), where *d*_2_ is the coefficient of the product term *w*_2_*m*_2_. Nevertheless, if we denote the effect from *x*_1_ to *m*_1_ with *w*_1_ = *w*_1∗_ by *a*_11∗_, and the effect from *m*_2_ to *y*_1_ with *w*_2_ = *w*_2∗_ by *b*_21∗_, then the conditional indirect effect in *x*_1_ → *m*_1_ → *m*_2_ → *y*_1_ when *w*_1_ = *w*_1∗_ and *w*_2_ = *w*_2∗_ can again be estimated by a product of path coefficients: *a*_11∗_*b*_12_*b*_21∗_. This applies to any other moderated paths in the model. For example, the path *x*_2_ → *m*_1_ → *m*_2_ → *y*_1_ only has one component path moderated: *m*_2_ → *y*_1_. The conditional indirect effect of this path when *w*_2_ = *w*_2∗_ is simply *a*_21_*b*_12_*b*_21∗_. Again, if meaningful, the conditional effect in *m*_1_ → *m*_2_ → *y*_1_ can be similarly estimated.

The *standardized conditional indirect effect* and *partially standardized conditional indirect effect* are the conditional indirect effects multiplied and divided by the corresponding *SD*s. For example, the standardized conditional indirect effect in the path *x*_1_ → *m*_1_ → *m*_2_ → *y*_1_ when *w*_1_ = *w*_1∗_ and *w*_2_ = *w*_2∗_ is ${a}_{11\ast }{b}_{12}{b}_{21\ast}\left(S{D}_{x_1}/S{D}_{y_1}\right)$. The partially standardized conditional indirect effect with *y*_1_ standardized, when *w*_1_ = *w*_1∗_ and *w*_2_ = *w*_2∗_, is ${a}_{11\ast }{b}_{12}{b}_{21\ast }/S{D}_{y_1}$.

Nonparametric percentile bootstrap CIs can also be formed for conditional indirect effects and standardized conditional indirect effects. For *R* bootstrap samples, the path coefficients, including those of the product terms, are estimated and then the conditional indirect effect is computed. The percentiles of these bootstrap estimates of the conditional indirect effect are then used to form the CI. For standardized and partially standardized conditional indirect effects, the corresponding *SD*s are also estimated in each bootstrap sample to compute the bootstrap estimates of the standardized or partially standardized conditional indirect effect for forming the CI.

## The two-stage workflow of *manymome*

Some common tools for mediation, moderation, and moderated mediation require users to perform parameter estimation and estimation of indirect and conditional indirect effects in one step (e.g., PROCESS and most SEM programs). This one-stage workflow, despite its convenience, has its limitations. First, researchers may be restricted by this tool with regard to the model that can be estimated and on aspects such as the numbers of predictors, outcome variables, mediators, moderators, or the form of the model. Users may need to find a workaround to use these tools. For example, the indirect effects and conditional indirect effects in Fig. 1 cannot be directly estimated by PROCESS in one step. Users need to call PROCESS several times to estimate all the parameters and effects. If one more moderator is added to the path from *x*_2_ to *y*_2_, or from *m*_1_ to *m*_2_, then PROCESS cannot be used at all because it does not support a model with more than two moderators (at the time of writing). Some other tools dedicated to mediation or moderation also have these limitations (e.g., *mediate()* in the *mediation* package, Tingley et al., 2014). This limitation can be overcome readily by SEM using *lavaan* (Rosseel, 2012), *Amos* (Arbuckle, 2021), or *M*plus (Muthén & Muthén, 2017). As long as researchers know how to define an effect, most indirect and conditional indirect effects, standardized or not, can be estimated by them.

However, this leads to the second limitation. Most SEM programs require users to define their own parameters for these effects, except for simple cases such as indirect effects, and only in some programs. For example, in *lavaan*, researchers need to define a parameter for every indirect effect before estimating a model. For conditional indirect effects, researchers also need to define all conditional indirect effects, including the values of the moderators, in advance (Miles et al., 2015). This means that, to explore a path or a level of moderator not defined in the model specification, the model syntax needs to be modified and the model refitted, which can be time-consuming when bootstrapping is required, and it can take even more time if missing data are also involved. Some programs, such as *Amos*, can enumerate all indirect effects automatically and report them, so that users do not need to decide which one to examine when specifying a model. However, when there is more than one path from one variable to another, *Amos* still requires researchers to define their own estimand for each path (Amos Development Corporation, 2021). For conditional indirect effects, especially standardized conditional indirect effects, the coding requirement is more demanding and error-prone because researchers need to know how to compute the *SD* of the outcome variable, which is not a free parameter in a model.^3^ Users can “trick” the program to give the standardized conditional indirect effects by standardizing the variables before fitting a model. However, as shown by Cheung (2009), standardizing the variables before fitting the model in these SEM programs to form the bootstrap CIs for indirect effects, called *naïve bootstrapping* by Cheung, will yield incorrect bootstrap CIs (see also Cheung et al., 2022, on standardization in moderation).

As illustrated earlier, parameter estimation and bootstrapping do not depend on the indirect effects to be estimated and the levels of the moderators on which the conditional indirect effects are conditional. Therefore, to address some of the limitations in the one-stage workflow, a two-stage workflow can be used: estimate all parameters and *SD*s first in Stage 1, with bootstrapping if necessary, and use the estimates to compute and test any indirect or conditional indirect effects in Stage 2. Some tools for mediation and moderation have already adopted this workflow. For example, the package *mediation* (Tingley et al., 2014) supports using the output of a wide variety of functions for regression and similar models. Researchers can use these tools to estimate the parameters and then use *mediate()* in the package to test mediation or moderated mediation. Our goal in *manymome* is to adopt this approach to support both SEM and multiple regression for models commonly fitted in behavioral research that involves mediation, moderation, and/or moderated mediation, and form the bootstrap CI appropriately for standardized effects as described by Cheung (2009).

In Stage 1 of *manymome*, users can estimate all parameters in a model using SEM (using *sem()* in *lavaan*) or regression (using *lm()*), without the need to code in advance the indirect effects and/or conditional indirect effects to be tested and the levels of the moderators, if any. This allows users to focus on model building and selection and avoid the hassle of defining indirect effects or conditional indirect effects in SEM. If SEM is used, there is no need to define any user parameters manually in specifying the model, and no need to label those parameters (see the examples in Miles et al., 2015). If bootstrap CI is needed, bootstrapping is done only once either in Stage 1 manually by users or automatically in the first run in Stage 2, with all model parameter estimates and other necessary information (e.g., *SD*s and means of variables) in all bootstrap samples stored. These bootstrap estimates of the model can then be reused in subsequent computation of any indirect and conditional indirect effects, without the need to perform the bootstrapping again.^4^

In Stage 2 of *manymome*, users call functions for indirect effects (*indirect_effect()*) and conditional indirect effects (*cond_indirect_effects()*) to compute the indirect effects along any path or conditional indirect effects along any path for any levels of the moderators using the estimates from Stage 1. This stage can be repeated as many times as necessary, without the need to fit the model or perform the bootstrapping again. Therefore, as long as researchers do not change the model, Stage 1 only needs to be conducted once. The two functions only need to know the path: where it starts (the predictor); where it ends (the outcome variable); and which variables it moves along (the mediators, if any), and the moderators (if any). They will then compute the requested indirect effects or conditional indirect effects as presented above using the parameter estimates.

### Supported stage 1 tools

The two-stage workflow allows us to develop a tool that leverages the features of existing tools for parameter estimation and model fitting in Stage 1, without the need to reinvent the wheel or develop tools that restrict the types of models supported. We decided to support *lavaan* when SEM is used in Stage 1 for two reasons. First, we believe it is one of the most popular SEM packages in R (R Core Team, 2022) and it supports a wide variety of methods for parameter estimation, model testing, standard errors, and model comparison. Second, researchers can fully utilize all available features in *lavaan* that they need for fitting a model and estimating its parameters. Because bootstrap CIs are used for indirect and conditional indirect effects, the estimation method used in SEM, which mainly affects the standard errors, does not affect the results in Stage 2 (as long as the parameter estimates are unbiased or not severely biased).

For Stage 1 estimation performed by regression, we decided to support *lm()* because it is usually the first function researchers learn for performing multiple regression when they learn R, and it is simple to use and readily supports moderators, continuous or categorical (just enter x*w, and the necessary product term and lower-order terms will be included in the model). Although *lm()* is rarely used for path analysis, regression-based tools like PROCESS actually perform a sequence of regression analysis as *lm()* does to estimate the parameters. Unlike other tools, users do not need to specify the predictor, mediators, and outcome variables in fitting these regression models by *lm()*. Just fit the necessary regression models and store the results. The functions in *manymome* will automatically identify the regression coefficients from the models for the indirect or conditional indirect effects along any path requested.

## Numerical examples

We will first illustrate the workflow in using *manymome* with several numerical examples. We will then discuss the strengths and current limitations of *manymome*. All the files used in the examples can be downloaded from the OSF page for this manuscript https://osf.io/mfkzg/). The package is available at the Comprehensive R Archive Network (CRAN) and can be installed by *install.packages("manymome")*.

### A model with both moderated mediation and mediation analyzed by SEM, with missing data

We do not follow the common practice of starting with simple models, as there are already numerous tools and illustrations for them. We use Model 1 for illustration because (a) the principle of the computation is not so different from that in simpler models, (b) it includes mediation, moderation, and moderated mediation in one model, (c) it shows the advantage of the two-stage workflow for an SEM model, and (d) it is fitted to a dataset with missing data to show how missing data can be handled. The indirect effect and conditional indirect effects in this model, though not uncommon, cannot be easily estimated and tested by various common tools because of (1) the form of the model, (2) the difficulty in coding, and/or (3) the presence of missing data.

The dataset to be used is *data_mome_demo_missing*, which comes with *manymome*. This dataset has all the variables in the conceptual models, plus two control variables (*c1* and *c2*). The number of cases is 200, with 31 cases having missing data. Therefore, if listwise deletion is used, about 16% of cases need to be removed, resulting in reduced statistical power.

#### Stage 1: Model fitting

If performed in SEM, this stage does not involve *manymome*. Researchers simply need to specify the desired model as usual, with no need to label any parameters or define any parameters. We follow the advice by Kwan and Chan (2018) and mean-center all variables first and then compute the product terms:^5^# Mean-center all variablesdata_centered <- as.data.frame(scale(data_mome_demo_missing, scale=FALSE))# Compute the Product Termsdata_centered$w1x1 <- data_centered$w1 * data_centered$x1data_centered$w2m2 <- data_centered$w2 * data_centered$m2

Next, this is the model syntax:mod <- "m1 ~ x1 + w1 + w1x1 + x2 + c1 + c2m2 ~ m1 + c1 + c2m3 ~ x2 + x1 + c1 + c2y1 ~ m2 + w2 + w2m2 + x1 + x2 + m3 + c1 + c2y2 ~ m3 + x2 + x1 + m2 + c1 + c2# Covariances for the error term of m2m2 ~~ w2 + w2m2# Covariances between all exogenous variablesw2 ~~ w2m2 + x1 + w1 + w1x1 + x2 + c1 + c2w2m2 ~~ x1 + w1 + w1x1 + x2 + c1 + c2x1 ~~ w1 + w1x1 + x2 + c1 + c2w1 ~~ w1x1 + x2 + c1 + c2w1x1 ~~ x2 + c1 + c2x2 ~~ c1 + c2c1 ~~ c2"

A few notes on the syntax. First, there is no need to label the path coefficients when specifying the path models. Stage 2 functions automatically identify the coefficients needed. Second, for the moderation by *w*_2_ on the path *m*_2_ → *y*_1_, the covariance between the error term of *m*_2_ and *w*_2_ and that between this error term and the product term *w*_2_*m*_2_ are included to account for the endogeneity of this product term (Kwan & Chan, 2018; Miles et al., 2015). Third, the seven lines on covariances are needed because *lavaan* will not add them automatically if we covary the error term with an exogenous variable (*w*_2_ in this model). This is a behavior of *lavaan* rather than of *manymome*. If researchers judge that covariances can be fixed to zero and so remove the line *m2 ~~ w2 + w2m2*, then all the lines on covariances can be removed because they will then be included automatically. Researchers can also use the VS program by Kwan and Chan (2018) to generate the model syntax. VS can generate all necessary covariances automatically. If only paths from exogenous variables to mediators are moderated, there is no need to covary an error term with a product term. For example, if only the path *x*_1_ → *m*_1_ is moderated, the syntax can be simplified as follows:mod_w1x1_only <- "m1 ~ x1 + w1 + w1x1 + x2 + c1 + c2m2 ~ m1 + c1 + c2m3 ~ x2 + x1 + c1 + c2y1 ~ m2 + x1 + x2 + m3 + c1 + c2y2 ~ m3 + x2 + x1 + m2 + c1 + c2"

The model can then be fitted by *sem()* in *lavaan*:library(lavaan)fit <- sem(mod, data_centered, fixed.x = FALSE, missing = "fiml")

Missing data are handled by full information maximum likelihood (FIML, Arbuckle, 1996). The argument *fixed.x =* *FALSE* is added such that the predictors and moderators are also treated as random variables, which usually make more sense in behavioral research. Nevertheless, there are also situations in which these variables, which are exogenous variables ("pure predictors") in this model, should be treated as fixed, such that they do not need be multivariate normal in the population (FIML assumes multivariate normality, see Enders, 2022). The functions in *manymome* handle the argument *fixed.x* in the same way as *lavaan* does, so researchers can set this option according to their situations. Interested readers can refer to Kline (2023, p. 136) for a discussion on this setting.

The model fits satisfactorily (model *χ*^2^[20] = 30.52, *p* = .062, comparative fit index [CFI] = .976, root mean square error of approximation [RMSEA] = .051). The parameter estimates are presented in Table 1. All the component paths in *x*_2_ → *m*_3_ → *y*_2_ are significant. All component paths in *x*_1_ → *m*_1_ → *m*_2_ → *y*_1_ are also significant. However, two of the component paths, *x*_1_ → *m*_1_ and *m*_2_ → *y*_1_, are moderated, and so conditional indirect effects need to be computed to examine how the indirect effect is affected by the moderators *w*_1_ and *w*_2_.

**Table 1:** Parameter estimates of Model 1

Predictor	Estimate	SE	*p*
Outcome: *m1*			
*x1*	0.352	0.072	.000***
*w1*	0.022	0.067	.743
*w1x1*	0.151	0.071	.033*
*x2*	0.158	0.061	.010*
Outcome: *m2*			
*m1*	0.548	0.057	.000***
Outcome: *y1*			
*m2*	0.826	0.142	.000***
*w2*	0.429	0.148	.004**
*w2m2*	0.801	0.091	.000***
*x1*	−0.031	0.149	.833
*x2*	−0.107	0.144	.457
*m3*	0.106	0.157	.498
Outcome: *m3*			
*x2*	0.495	0.055	.000***
*x1*	0.321	0.063	.000***
Outcome: *y2*			
*m3*	0.550	0.067	.000***
*x2*	−0.016	0.061	.789
*x1*	0.029	0.063	.643
*m2*	−0.033	0.055	.551

One note on this stage. Although researchers can request bootstrap CI in this stage using *se = "boot"*, this is not necessary, as illustrated next. Researchers can use whatever method for standard errors and CIs as appropriate for the model and the data (e.g., robust standard error by MLR for nonnormal variable) in estimating and testing the model parameters.

#### Stage 2: Indirect effects and conditional indirect effects

##### Indirect effects

To estimate the indirect effect along a path not moderated, we can use *indirect_effect()*. This is the call to estimate the indirect effect in *x*_2_ → *m*_3_ → *y*_2_:out_ind <- indirect_effect(x = "x2", y = "y2", m = "m3", fit = fit, boot_ci = TRUE, R = 5000, ncores = 9, seed = 43143)

The argument *x* is the name of the variable at the start of the path. The argument *y* is the name of the variable at the end of the path. The argument *m* is a vector of the names of mediators in the path, ordered from the start to the end of the path. This example has only one mediator, *m3*. The argument *fit* is the output of *sem()* from *lavaan*, created in Stage 1.

To request bootstrapping CI, set *boot_ci* to *TRUE*, *R* to the number of bootstrap samples^6^, and *seed* to an arbitrary integer for reproducible results.^7^ In the current version, only nonparametric bootstrapping is supported. By default, parallel processing will be used, and the number of processes is equal to the number of physical CPU cores minus 1. The number of processes can be set manually by setting *ncores* to the desired number of processes. We decided to enable parallel processing by default because bootstrapping can be slow when used with missing data. When it was run on a computer with an Intel Core i7-8700 CPU without parallel processing, this example took 8 to 9 minutes. With parallel processing and nine processes (*ncores = 9*), it took only about 1 to 2 minutes. Given that many contemporary computers have more than one core, it is safe to enable this by default. Researchers can disable parallel processing by setting *parallel* to *FALSE*. As noted, this bootstrapping step only needs to be done once because all parameter estimates and *SD*s will be stored in the output, regardless of the path being tested. The results can be reused in subsequent calls, to be illustrated later, so that there is no need to perform bootstrapping again.

This is an excerpt of the output when it is printed> out_ind== Indirect Effect == Path: x2 -> m3 -> y2 Indirect Effect 0.272 95.0% Bootstrap CI: [0.188 to 0.366]Computation Formula: (b.m3~x2)*(b.y2~m3)Computation: (0.49472)*(0.55018)Coefficients of Component Paths: Path Coefficient m3~x2 0.495 y2~m3 0.550

The indirect effect in *x*_2_ → *m*_3_ → *y*_2_ is 0.272, 95% bootstrap CI 0.188–0.366, significant at *p* < .05. For understanding and verifying the results, it also prints the component paths involved and how the indirect effect is computed. If only the indirect effect estimate and the CI are needed, they can be extracted by the generic functions *coef()* and *confint()*:> coef(out_ind) y2~x20.2721852> confint(out_ind) 2.5 % 97.5 %y2~x2 0.1880765 0.3657727

Therefore, researchers can call *indirect_effect()* and then retrieve the estimate and confidence interval for other purposes.

Suppose we also want to estimate the indirect effect in *x*_2_ → *m*_1_ → *m*_2_ → *y*_2_. The call is nearly the same:out_ind2 <- indirect_effect(x = "x2", y = "y2", m = c("m1", "m2"), fit = fit, boot_ci = TRUE, boot_out = out_ind)

There are two major differences. First, the value of the argument *m* is *c("m1", "m2")*. This denotes the path *x*_2_ → *m*_1_ → *m*_2_ → *y*_2_. There is no known limit on the number of mediators in *manymome*. The function will automatically identify the component paths and their coefficients from the model. It will also check the validity of this path first to prevent misspecification. Therefore, setting *m* to *"m1"* will only result in an error message saying that the path *x*_2_ → *m*_1_ → *y*_2_ does not exist in the model. Setting *m* to *c("m2", "m1")* will also result in error because the direction is wrong. Second, the argument *boot_out* is used and set to *out_ind*, and the output from the previous call to *indirect_effect()* (or *cond_indirect_effects()*, presented next). The function will check whether bootstrap estimates are available in the value of *boot_out*, and if yes, will reuse them without performing bootstrapping again to form the bootstrap CI. Therefore, this step, while still needing to perform the calculation 5000 times, only took about 2 seconds on the same computer, versus 1 to 2 minutes in the previous call. Researchers can also save the output with bootstrap estimates (*out_ind* in this example) to a file by *saveRDS()* or *save()* and load it later to explore other indirect effects, without doing the bootstrapping again, as long as the model does not change.

Two remarks on using *indirect_effect()*. First, it can be used to estimate and test the indirect effect along *any* path in a model. Therefore, if we want to test the indirect effect in *m*_1_ → *m*_2_ → *y*_2_, just set *x* to *"m1"*, *y* to *"y2"*, and *m* to *"m2"*. For a complicated model, researchers can freely explore any path without respecifying and refitting the model, which can be time-consuming with bootstrapping and missing data. Second, the function will automatically check whether any component path has one or more moderators. Therefore, if we set *x* to *"x1"*, *y* to *"y1"*, and *m* to *c("m1", "m2")*, denoting the path *x*_1_ → *m*_1_ → *m*_2_ → *y*_1_, it will give the estimates of the indirect effects but will also issue a warning that one or more component paths have one or more moderators (*x*_1_ → *m*_1_ and *m*_2_ → *y*_1_ in this example). The seemingly unconditional indirect effect is actually the indirect effect when all these moderators are equal to zero. It is the responsibility of the researchers to decide whether this indirect effect is interpretable.

##### **Conditional indirect effects**

To estimate and test a conditional indirect effect, the function *cond_indirect_effects()* can be used. This is a call to estimate the conditional indirect effects in *x*_1_ → *m*_1_ → *m*_2_ → *y*_1_:out_cond <- cond_indirect_effects(wlevels = c("w1", "w2"), x = "x1", y = "y1", m = c("m1", "m2"), fit = fit, boot_ci = TRUE, boot_out = out_ind)

The arguments *x*, *y*, *m*, *fit*, *boot_ci*, and *boot_out* are specified in the same way as in *indirect_effect()*. Note that the bootstrap estimates in *out_ind*, though created when we estimate an indirect effect, can still be reused here because the model parameter estimates do not depend on the effects being tested. The new argument, *wlevels*, accepts several forms of input. If the default behavior is sufficient, then researchers can simply set it to the variable names of the moderators along the path, *c("w1", "w2")* in this example. There is no need to specify which paths they moderate. The function will automatically identify paths they moderate and compute the conditional effect accordingly, even if a moderator moderates more than one path.^8^ Advanced uses of this argument, for example, for categorical moderators represented by two or more dummy variables, are presented later.

If the first call that involves bootstrapping is *cond_indirect_effects()*, then the argument *R*, *ncores*, and *seed* can also be set as in *indirect_effect()*.out_cond <- cond_indirect_effects(wlevels = c("w1", "w2"), x = "x1", y = "y1", m = c("m1", "m2"), fit = fit, boot_ci = TRUE, R = 5000, ncores = 9, seed = 43143)

Once the bootstrapping is conducted, the estimates will be stored in the output of *cond_indirect_effects()*. The stored estimates can be used by other calls to *cond_indirect_effects()* and *indirect_effect()*.

This is an excerpt of the results when the output of *cond_indirect_effects()* is printed:^9^== Conditional indirect effects == Path: x1 -> m1 -> m2 -> y1 Conditional on moderator(s): w1, w2 [w1] [w2] (w1) (w2) ind CI.lo CI.hi Sig m1~x1 m2~m1 y1~m21 M+1.0SD M+1.0SD 1.011 0.959 0.441 0.225 0.703 Sig 0.505 0.548 1.5942 M+1.0SD M-1.0SD 1.011 -0.959 0.016 -0.065 0.103 0.505 0.548 0.0583 M-1.0SD M+1.0SD -1.011 0.959 0.175 -0.015 0.389 0.200 0.548 1.5944 M-1.0SD M-1.0SD -1.011 -0.959 0.006 -0.027 0.047 0.200 0.548 0.058 - [CI.lo to CI.hi] are 95.0% percentile confidence intervals by nonparametric bootstrapping with 5000 samples. - The 'ind' column shows the indirect effects. - 'm1~x1','m2~m1','y1~m2' is/are the path coefficient(s) along the path conditional on the moderators.

By default, if there are more than two moderators, two levels of each continuous moderator will be used: 1 *SD* below mean and 1 *SD* above mean. For two continuous moderators, the number of combinations is 4. These default levels can be overridden (presented later). The printout reports the indirect effect conditional on each combination of the moderators, along with the bootstrap CI, if requested. For understanding and verifying the results, the component paths conditional on each combination are also printed (using *get_one_cond_indirect_effect()*, described later). As expected, the path *m*_1_ → *m*_2_ (*m2~m1* in *lavaan* syntax) is the same for all combinations because it is not moderated. If either *w*_1_ or *w*_2_ increases, the indirect effect increases because the effect in either *x*_1_ → *m*_1_ or *m*_2_ → *y*_1_ increases. However, for the four levels examined, the indirect effect is significant only for the condition with both *w*_1_ and *w*_2_ 1 *SD* above their means.

Like *indirect_effect()*, *cond_indirect_effects()* can be used to examine the conditional indirect effect for any path in a model by setting *x*, *y*, and *m* accordingly. For example, this call estimates the effect in *x*_1_ → *m*_1_ → *m*_2_ → *y*_2_, moderated only by *w*_1_:out_cond <- cond_indirect_effects(wlevels = "w1", x = "x1", y = "y2", m = c("m1", "m2"), fit = fit, boot_ci = TRUE, boot_out = out_ind)

This is an excerpt of the output:Path: x1 -> m1 -> m2 -> y2 Conditional on moderator(s): w1 [w1] (w1) ind CI.lo CI.hi Sig m1~x1 m2~m1 y2~m21 M+1.0SD 1.011 -0.009 -0.041 0.022 0.505 0.548 -0.0332 Mean 0.000 -0.006 -0.030 0.015 0.352 0.548 -0.0333 M-1.0SD -1.011 -0.004 -0.023 0.008 0.200 0.548 -0.033

With only one moderator, three levels will be used by default for continuous moderator: 1 *SD* below mean, mean, and 1 *SD* above mean. The indirect effects for all three levels are not significant, suggesting that in this range (1 *SD* within mean of *w*_1_), there is no significant indirect effect from *x*_1_ to *y*_2_ along this path. The printout also shows that, as expected, only one of the component paths (*m1~x1*) depends on *w*_1_.^10^

##### Standardized effects

To estimate and test the standardized or partially standardized indirect effect or conditional indirect effect, *indirect_effect()* and *cond_indirect_effects()* can be used, with either the argument *standardized_x* or *standardized_y* set to *TRUE* for partially standardized effects and both set to *TRUE* for (completely) standardized effects. For example, this call is for the standardized indirect effect in *x*_2_ → *m*_3_ → *y*_2_:out_ind_std <- indirect_effect(x = "x2", y = "y2", m = "m3", fit = fit, boot_ci = TRUE, boot_out = out_ind, standardized_x = TRUE, standardized_y = TRUE)

This is an excerpt of the output: Path: x2 -> m3 -> y2 Indirect Effect 0.305 95.0% Bootstrap CI: [0.220 to 0.393]Computation Formula: (b.m3~x2)*(b.y2~m3)*sd_x2/sd_y2Computation: (0.49472)*(0.55018)*(1.04844)/(0.93431)NOTE: The effects of the component paths are from the model, not standardized.

The standardized indirect effect is 0.305, with 95% CI 0.220–0.393, significant (*p* < .05). The computation formula shows the *SD*s it used to standardize the indirect effect. Note that, as discussed before, all four numbers, the two path coefficients and the two *SD*s, are estimated in each bootstrap sample, the *SD* of the outcome variable being the model-implied *SD* because it is not a model parameter. The generic functions *coef()* and *confint()* can be used to extract the estimate and the confidence interval:> coef(out_ind_std) y2~x20.305434> confint(out_ind_std) 2.5 % 97.5 %y2~x2 0.2197278 0.3933275

This is the call to estimate the standardized conditional indirect effect in *x*_1_ → *m*_1_ → *m*_2_ → *y*_1_, moderated by *w*_1_ and *w*_2_:out_cond_std <- cond_indirect_effects(wlevels = c("w1", "w2"), x = "x1", y = "y1", m = c("m1", "m2"), fit = fit, boot_ci = TRUE, boot_out = out_ind, standardized_x = TRUE, standardized_y = TRUE)

This is an excerpt of the output: Path: x1 -> m1 -> m2 -> y1 Conditional on moderator(s): w1, w2 Moderator(s) represented by: w1, w2 [w1] [w2] (w1) (w2) std CI.lo CI.hi Sig m1~x1 m2~m1 y1~m2M+1.0SD M+1.0SD 1.011 0.959 0.173 0.089 0.282 Sig 0.505 0.548 1.594M+1.0SD M-1.0SD 1.011 -0.959 0.006 -0.027 0.040 0.505 0.548 0.058M-1.0SD M+1.0SD -1.011 0.959 0.069 -0.007 0.150 0.200 0.548 1.594M-1.0SD M-1.0SD -1.011 -0.959 0.003 -0.011 0.018 0.200 0.548 0.058 - std: The standardized indirect effects.

The standardized conditional effects for the four levels are reported in the column *std*. The unstandardized component paths are also reported. As noted, computing the standardized conditional indirect effect only needs to multiply and/or divide the conditional indirect effect by the *SD*s of the *x* variable and *y* variable, and there is no need to standardize each component path in the computation. Consistent with what we found before, for the four levels examined, only at the level where both *w*_1_ and *w*_2_ are 1 *SD* above their means is the standardized indirect effect in *x*_1_ → *m*_1_ → *m*_2_ → *y*_1_ (0.173) significant (*p* < .05).

##### Moderation only

Although our focus is on mediation and moderated mediation, *cond_indirect_effects()* can also be used to estimate a path with only moderation, which can be useful to visualize the moderation effect of a component path in a path with mediators .^11^ For example, to visualize the moderation of *w*_1_ and *w*_2_ on paths *x*_1_ → *m*_1_ and *m*_2_ → *y*_1_, respectively, we can use *cond_indirect_effects()* without the *m* argument:out_cond_x1m1 <- cond_indirect_effects(wlevels = c("w1"), x = "x1", y = "m1", fit = fit, boot_ci = TRUE, boot_out = out_cond)out_cond_m2y1 <- cond_indirect_effects(wlevels = c("w2"), x = "m2", y = "y1", fit = fit, boot_ci = TRUE, boot_out = out_cond)

These are part of the outputs: Path: x1 -> m1 Conditional on moderator(s): w1 [w1] (w1) ind CI.lo CI.hi Sig m1~x11 M+1.0SD 1.011 0.505 0.296 0.716 Sig 0.5052 Mean 0.000 0.352 0.207 0.500 Sig 0.3523 M-1.0SD -1.011 0.200 -0.017 0.424 0.200 Path: m2 -> y1 Conditional on moderator(s): w2 [w2] (w2) ind CI.lo CI.hi Sig y1~m21 M+1.0SD 0.959 1.594 1.288 1.906 Sig 1.5942 Mean 0.000 0.826 0.552 1.089 Sig 0.8263 M-1.0SD -0.959 0.058 -0.232 0.324 0.058

The *plot()* generic function can be used to plot the conditional effects:plot(out_cond_x1m1)plot(out_cond_m2y1)

**Fig. 2:**
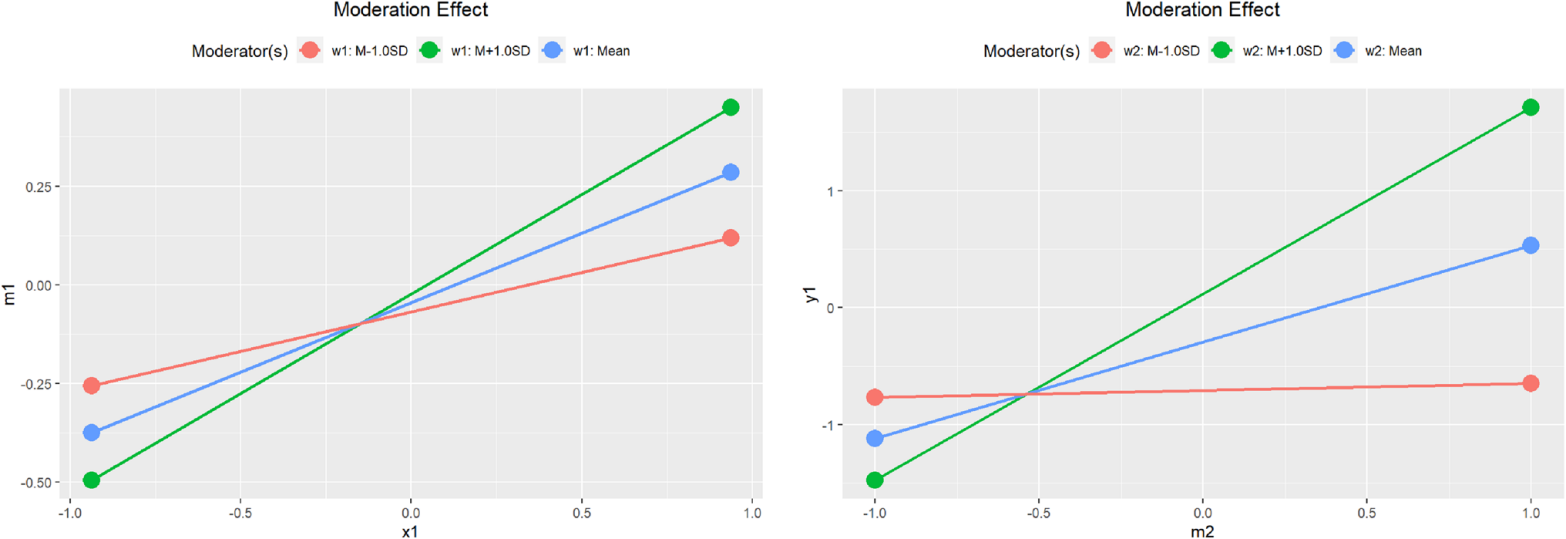
Model 1: Plots of moderation effects

The plots are shown in Fig. 2.

### A moderated mediation model with one categorical moderator and one continuous moderator, with missing data

**Fig. 3:**
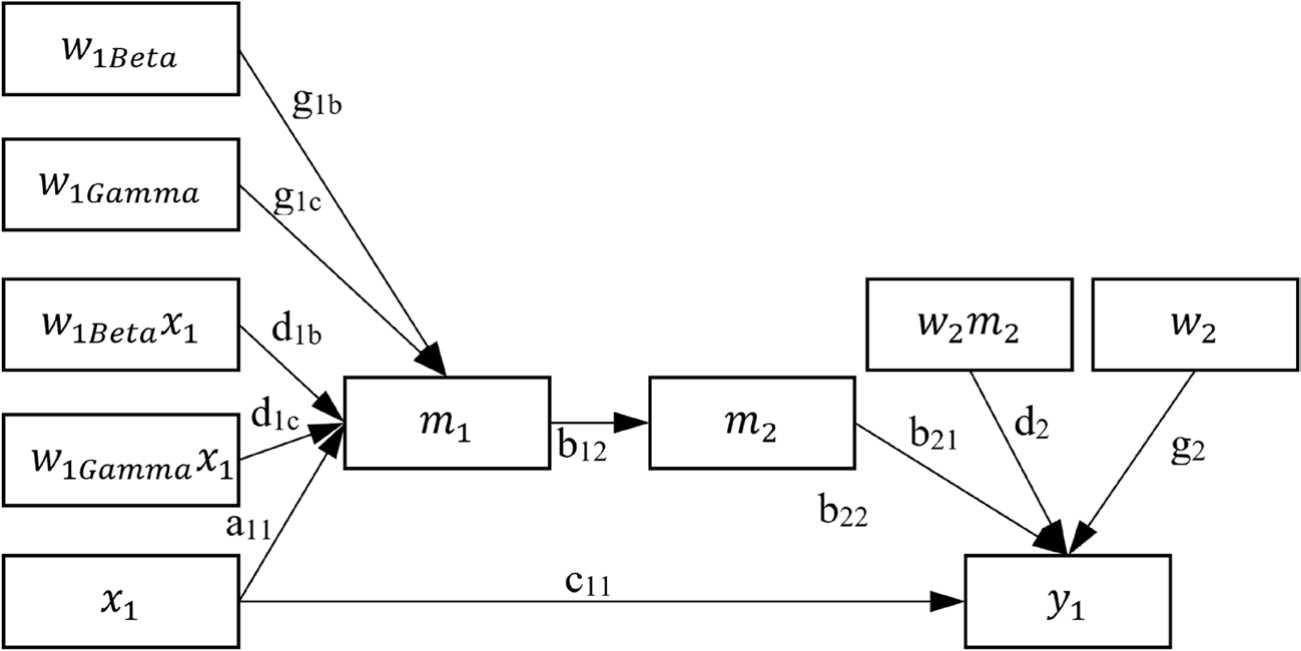
Model 2: The working model, W_1Beta_ and W_1Gamma_ the dummy variables of the categorical moderator W_1_ (variances and covariances omitted for readability)

To illustrate how *manymome* can also be used for a model with categorical moderators, we use the same dataset, *data_mome_demo_missing*, and (a) convert *w*_1_ to a three-group categorical variable for the sake of illustration, and (b) fit Model 2, a subset of Model 1 with only the path from *x*_1_ to *y*_1_ (Fig. 3).

#### Stage 1: Model fitting

Using the mean centered dataset in the previous example, we create *w1gp*, a column of a categorical variable with three groups, *Alpha*, *Beta*, and *Gamma*, using the cutoff values −0.4 and 0.4 to obtain three groups with similar group sizes:data_centered$w1gp <- cut(data_centered$w1, breaks = c(-Inf, -.4, .4, Inf), labels = c("Alpha", "Beta", "Gamma"))

The *lavaan* package does not support using categorical variables directly. We need to create the two dummy variables manually. Researchers can do this themselves. The *manymome* package also provides a basic function, *factor2var()*, to do this:data_centered[c("w1Beta", "w1Gamma")] <- factor2var(data_centered$w1gp, prefix = "w1")

The first argument is the vector of the categorical variable (*data_centered$w1gp*). Alternatively, we can provide a prefix to the dummy variables to be created by setting *prefix*. Because two dummy variables will be created, *c("w1Beta", "w1Gamma")* is used to add them to the data frame. These are the columns created (for the first six observations).> head(data_centered[, c("w1gp", "w1Beta", "w1Gamma")]) w1gp w1Beta w1Gamma1 Alpha 0 02 Beta 1 03 Alpha 0 04 Gamma 0 15 Alpha 0 06 Gamma 0 1

After confirming that the dummy variables are correctly created, we can create the product terms between them and *x*_1_:data_centered$w1Betax1 <- data_centered$w1Beta * data_centered$x1data_centered$w1Gammax1 <- data_centered$w1Gamma * data_centered$x1data_centered$w2m2 <- data_centered$w2 * data_centered$m2

This is the model syntax:mod <- "m1 ~ x1 + w1Beta + w1Gamma + w1Betax1 + w1Gammax1+ c1 + c2m2 ~ m1 + c1 + c2y1 ~ m2 + w2 + w2m2 + x1 + c1 + c2# Covariances for the error term of m2m2 ~~ w2 + w2m2# Covariances between all exogenous variablesw2 ~~ w2m2 + x1 + w1Beta + w1Gamma + w1Betax1 + w1Gammax1 + c1 + c2w2m2 ~~ x1 + w1Beta + w1Gamma + w1Betax1 + w1Gammax1 + c1 + c2x1 ~~ w1Beta + w1Gamma + w1Betax1 + w1Gammax1 + c1 + c2w1Beta ~~ w1Gamma + w1Betax1 + w1Gammax1 + c1 + c2w1Gamma ~~ w1Betax1 + w1Gammax1 + c1 + c2w1Betax1 ~~ w1Gammax1 + c1 + c2w1Gammax1 ~~ c1 + c2c1 ~~ c2"

This is similar to the one in the previous example, with *x*_2_, *m*_3_, and *y*_2_ removed. We fit this model as before using FIML to handle missing data:^12^fit <- sem(mod, data_centered, fixed.x = FALSE, missing = "fiml")

The model fits satisfactorily (model *χ*^2^[12] = 22.10, *p* = .036, CFI = .983, RMSEA = .065). The parameter estimates are presented in Table 2. All component paths in *x*_1_ → *m*_1_ → *m*_2_ → *y*_1_ are significant except for *x*_1_ → *m*_1_ (0.196, *p* = .141). However, the product of *w*_1_*Gamma* and *x*_1_ is significant (.434, *p* = .024), suggesting that the path *x*_1_ → *m*_1_ is moderated by *w*_1_. The product of *w*_2_ and *m*_2_ is also significant (.799, *p* < .001). Therefore, the conditional indirect effects need to be computed along this path.

**Table 2:** Parameter estimates of Model 2

Predictor	Estimate	SE	*p*
Outcome: *m1*			
*x1*	0.196	0.133	0.141
*w1Beta*	−0.116	0.167	0.485
*w1Gamma*	−0.178	0.174	0.306
*w1Betax1*	0.125	0.176	0.478
*w1Gammax1*	0.434	0.192	0.024*
Outcome: *m2*			
*m1*	0.551	0.057	0.000***
Outcome: *y1*			
*m2*	0.820	0.142	0.000***
*w2*	0.439	0.148	0.003**
*w2m2*	0.799	0.091	0.000***
*x1*	0.005	0.140	0.970

#### Stage 2: Conditional indirect effects

In this model we focus on conditional indirect effects. We first examine the component path *x*_1_ → *m*_1_ to illustrate how to specify a categorical moderator represented by two or more dummy variables. We can use *cond_indirect_effects()* without setting *m*:out_cond <- cond_indirect_effects( wlevels = list(w1 = c("w1Beta", "w1Gamma")), x = "x1", y = "m1", fit = fit, boot_ci = TRUE, R = 5000, parallel = TRUE, ncores = 9, seed = 43143)

All arguments are specified as in Model 1, except for *wlevels*. To tell the function that *w1Beta* and *w1Gamma* together represent a single moderator called *w1*, we use a named list, with the name representing the name of the moderator (not in the model) and its element a character vector of the names of the dummy variables (*c("w1Beta", "w1Gamma")*) in the model. Although bootstrapping is not necessary for this analysis, requesting bootstrapping allows the estimates to be reused in subsequent analysis.

This is an excerpt of the printout:== Conditional effects == Path: x1 -> m1 Conditional on moderator(s): w1 Moderator(s) represented by: w1Beta, w1Gamma [w1] (w1Beta) (w1Gamma) ind CI.lo CI.hi Sig m1~x11 Reference 0 0 0.196 -0.079 0.449 0.1962 Beta 1 0 0.321 0.055 0.611 Sig 0.3213 Gamma 0 1 0.630 0.367 0.905 Sig 0.630

**Fig. 4:**
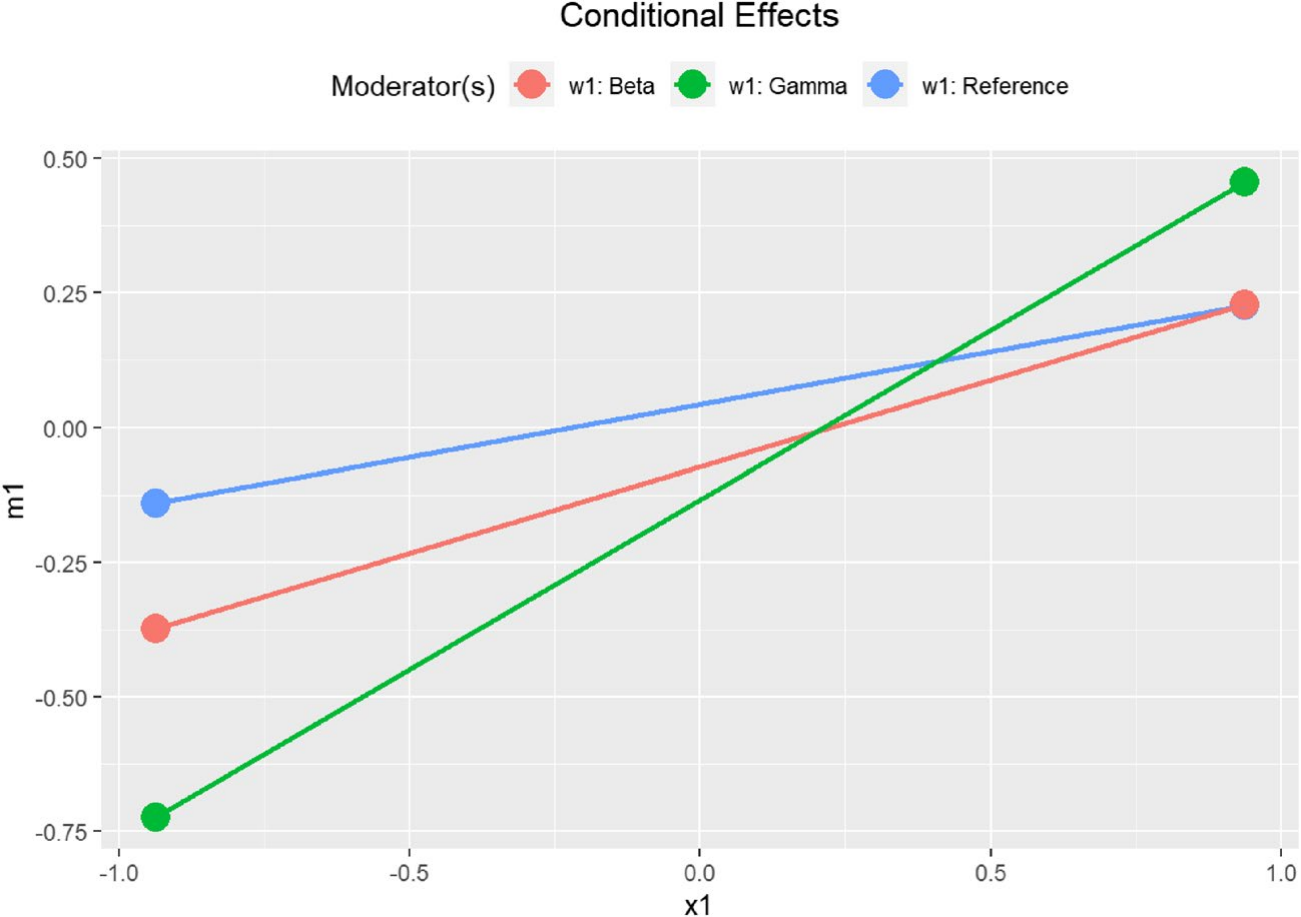
Model 2: Plots of moderation effects from X_1_ to M_1_

The function automatically enumerates the groups and tries to infer the group names from the dummy variable names.^13^ Based on the bootstrap CIs, the effect of *x*_1_ on *m*_1_ is positive and significant for the group *Beta* and *Gamma* but not for the reference group (*Alpha*). Figure 4 shows a plot of the conditional effects for these groups (using *plot(out_cond)*).

To compute the conditional indirect effects in *x*_1_ → *m*_1_ → *m*_2_ → *y*_1_, moderated by *w*_1_ (represented by *w1Beta* and *w1Gamma*) and *w*_2_, we use *cond_indirect_effects()* and reuse the bootstrap estimates:out_cond2 <- cond_indirect_effects( wlevels = list(w1 = c("w1Beta", "w1Gamma"), w2 = "w2"), x = "x1", y = "y1", m = c("m1", "m2"), fit = fit, boot_ci = TRUE, boot_out = out_cond)

There is one major change in setting *wlevels*. When at least one of the moderators is categorical, it needs to be a named list, where the names are the names of the moderators (not necessarily the variable names in the model) and the values are either (a) a vector of names of the dummy variables for a categorical moderator (*c("w1Beta", "w1Gamma")*) or (b) a single name of the variable of a continuous moderator (*"w2"*). All other arguments are set as in the previous example. This is an excerpt of the printout: Path: x1 -> m1 -> m2 -> y1 Conditional on moderator(s): w1, w2 Moderator(s) represented by: w1Beta, w1Gamma, w2 [w1] [w2] (w1Beta) (w1Gamma) (w2) ind CI.lo CI.hi Sig1 Reference M+1.0SD 0 0 0.959 0.171 -0.066 0.4232 Reference M-1.0SD 0 0 -0.959 0.006 -0.027 0.0533 Beta M+1.0SD 1 0 0.959 0.280 0.045 0.565 Sig4 Beta M-1.0SD 1 0 -0.959 0.009 -0.048 0.0715 Gamma M+1.0SD 0 1 0.959 0.551 0.271 0.894 Sig6 Gamma M-1.0SD 0 1 -0.959 0.019 -0.078 0.131

With two moderators, continuous moderators have two levels each by default, resulting in six combinations in this example. When all paths and moderators are considered, among the six combinations, the indirect effect is significant only when *w*_2_ is 1 *SD* above the mean and only in the groups *Beta* and *Gamma*. Therefore, although *x*_1_ → *m*_1_ is significant in both *Beta* and *Gamma* groups, the indirect effect along the path is not significant in these two groups when *w*_2_ is 1 *SD* below the mean.

### The index of moderated mediation and the index of moderated moderated mediation

Hayes (2015) proposed a useful measure of the effect of moderator in a moderated mediation model: the index of moderated mediation. When there is only one mediator, this is the change in the indirect effect when the moderator increases by one unit. When there are two moderators, Hayes (2018) also proposed the index of moderated moderated mediation, which is the change in the moderation of one moderator when the other moderator increases by one unit. The package *manymome* can directly compute these indices automatically using the function *index_of_mome()* and *index_of_momome()*.

#### The index of moderated mediation

The function *index_of_mome()* can be used to compute the difference in conditional indirect effects for a path with one moderator and one or more mediators. We use the path *m*_1_ → *m*_2_ → *y*_1_, moderated only by *w*_2_, for illustration, reusing the bootstrap estimates stored in *out_ind*:index_m1m2y1 <- index_of_mome(x = "m1", y = "y1", m = "m2", w = "w2", fit = fit, boot_ci = TRUE, boot_out = out_ind)

This is an excerpt of the output: Path: m1 -> m2 -> y1 Conditional on moderator(s): w2 Moderator(s) represented by: w2 [w2] (w2) ind CI.lo CI.hi Sig m2~m1 y1~m21 1 1 0.892 0.639 1.159 Sig 0.548 1.6272 0 0 0.453 0.278 0.640 Sig 0.548 0.826== Index of Moderated Mediation == x y Index CI.lo CI.hiIndex m1 y1 0.439 0.329 0.547

The moderator *w*_2_ increased from Row 2 to Row 1 by one unit, and the indirect effect along this path increases from 0.453 to 0.892. The difference, 0.439, is the index of moderated mediation of *w*_2_ for the path *m*_1_ → *m*_2_ → *y*_1_. The bootstrap CI is 0.329–0.547.

The arguments of *index_of_mome()* are similar to those in *cond_indirect_effects()*, but there is no need to specify the level of the moderator (*w*). There is no limit on the number of mediators (*m*), and there is no need to specify which path the moderator moderates.

#### Index of moderated moderated mediation

When a path has two moderators, *index_of_momome()* can be used to compute the index of moderated moderated mediation. For example, this index for the path *x*_1_ → *m*_1_ → *m*_2_ → *y*_1_, moderated by *w*_1_ and *w*_2_, can be found as follows:index_x1m1m2y1 <- index_of_momome(x = "x1", y = "y1", m = c("m1", "m2"), w = "w1", z = "w2", fit = fit, boot_ci = TRUE, boot_out = out_ind)

This is an excerpt of the output: Path: x1 -> m1 -> m2 -> y1 Conditional on moderator(s): w1, w2 Moderator(s) represented by: w1, w2 [w1] [w2] (w1) (w2) ind CI.lo CI.hi Sig m1~x1 m2~m1 y1~m21 1 1 1 1 0.449 0.229 0.715 Sig 0.503 0.548 1.6272 1 0 1 0 0.228 0.106 0.391 Sig 0.503 0.548 0.8263 0 1 0 1 0.314 0.162 0.496 Sig 0.352 0.548 1.6274 0 0 0 0 0.160 0.075 0.270 Sig 0.352 0.548 0.826== Index of Moderated Moderated Mediation ==Levels compared:(Row 1 - Row 2) - (Row 3 - Row 4) x y Index CI.lo CI.hiIndex x1 y1 0.066 -0.005 0.144

The index of moderated moderated mediation of *w*_1_ and *w*_2_ for the path *x*_1_ → *m*_1_ → *m*_2_ → *y*_1_ is 0.066, with bootstrap CI −0.005 to 0.144. Although the moderation effect of *w*_1_ on the indirect effect changes when *w*_2_ increases by one unit, and vice versa, the increase is not significantly different from zero.

The arguments of *index_of_momome()* are also similar to those in *index_of_mome()*, with one more argument, *z*, for the second moderator. There is no limit on the number of mediators (*m*), and there is no need to specify which path the two moderators moderate.

### Difference between any two conditional indirect effects

The function *cond_indirect_diff()* is a generalized version of *index_of_mome()* and *index_of_momome()* for computing and testing the difference between any two conditional indirect effects. To illustrate the case of one moderator, we compute the conditional indirect effects in *m*_1_ → *m*_2_ → *y*_1_, moderated only by *w*_2_, reusing the bootstrap estimates stored in *out_ind*:out_cond_m1y1 <- cond_indirect_effects(wlevels = "w2", x = "m1", y = "y1", m = "m2", fit = fit, boot_ci = TRUE, boot_out = out_ind)

This is an excerpt of the output: [w2] (w2) ind CI.lo CI.hi Sig m2~m1 y1~m21 M+1.0SD 0.959 0.874 0.625 1.136 Sig 0.548 1.5942 Mean 0.000 0.453 0.278 0.640 Sig 0.548 0.8263 M-1.0SD -0.959 0.032 -0.123 0.184 0.548 0.058

The moderator *w*_2_ increased from Row 2 to Row 1 by 1 *SD* (0.959), and the indirect effect along this path increases from 0.453 to 0.874. The indirect effects at these two levels of *w*_2_ are both significant. Suppose we are interested in the change in the indirect effect when the moderator (*w*_2_) increased by 1 *SD*; we can use the function *cond_indirect_diff()*:ind_diff <- cond_indirect_diff(out_cond_m1y1, from = 2, to = 1)

The first argument is the output of *cond_indirect_effects()*. The arguments *from* and *to* are the row numbers. In this example, *from = 2* and *to = 1* indicate that the change in the indirect effect from *w*_2_ equal to mean (Row 2) to *w*_2_ equal to 1 *SD* above mean (Row 1) is to be computed. This is an excerpt of the results:Levels: w2To: w2: M+1.0SD 0.959From: w2: Mean 0.000Change in Indirect Effect: x y Change CI.lo CI.hiIndex m1 y1 0.421 0.315 0.524

The change is 0.421 (0.874–0.453), with 95% bootstrap CI 0.315–0.524. The increase is significant, *p* < .05. Note that because the moderation effect is linear in the model, this change is the same for any interval with an increase of 1 *SD* for the moderator (e.g., from 1 *SD* below mean to mean). We denote this change in indirect effect for an increase of 1 *SD* in the moderator the *z-index of moderated mediation*. The *z*-index of moderated mediation is more interpretable when the moderator does not have a meaningful unit.

By setting the levels of the moderator to desired values (presented later), *cond_indirect_diff()* can be used to compute the difference between any two conditional indirect effects and form its confidence interval. If the moderator is categorical, then *cond_indirect_diff()* can be used to compute the difference in indirect effects between any two groups.

### Total effect, total indirect effect, or any other functions of effects

In some models, it may be of interest to compute a function of indirect or conditional indirect effects. For example, in Model 1, researchers may be interested in computing the *total indirect effect* from *x*_2_ to *y*_2_ through the two paths: *x*_2_ → *m*_3_ → *y*_2_ and *x*_2_ → *m*_1_ → *m*_2_ → *y*_2_. The output of *indirect_effect()*, which is an *indirect* class object, supports addition and subtraction, with bootstrap confidence intervals if they are also requested for the indirect effects used in the operation.^14^ Suppose the outputs of *indirect_effect()* for the two paths are *out_ind* and *out_ind2*, respectively; the total indirect effect can be computed as follows:out_ind_total <- out_ind + out_ind2

This is an excerpt of the output when printed: Path: x2 -> m3 -> y2 Path: x2 -> m1 -> m2 -> y2 Function of Effects: 0.269 95.0% Bootstrap CI: [0.184 to 0.363]Computation of the Function of Effects: (x2->m3->y2)+(x2->m1->m2->y2)

The output is similar to that for one indirect effect, with the paths being added listed. The total indirect effect is 0.269, 95% bootstrap CI 0.184–0.363, significant (*p* < .05).

The total effect from *x*_2_ to *y*_2_ is the sum of the effects for all paths, including the direct path *x*_2_ → *y*_2_. The direct effect can be computed by *indirect_effect()* with the argument *m* not set:out_direct <- indirect_effect(x = "x2", y = "y2", fit = fit, boot_ci = TRUE, boot_out = out_ind)

The total effect can be computed as the sum of all these effects:out_total <- out_ind + out_ind2 + out_direct

This is an excerpt of the output if printed:== Indirect Effect == Path: x2 -> m3 -> y2 Path: x2 -> m1 -> m2 -> y2 Path: x2 -> y2 Function of Effects: 0.253 95.0% Bootstrap CI: [0.138 to 0.367]

The total effect is 0.253, 95% bootstrap CI 0.138–0.367, significant (*p* < .05). It is not described here, but the output of *indirect_effect()* also supports the "−" (subtraction) operator, allowing researchers to compute and test the difference between the indirect effects along two different paths.

## Other options available

We limited the number of options used above to give readers a basic idea of how to use the functions. When necessary, there are other options available for researchers to enable greater control over the analysis. A few of them are introduced below.

### Customizing the levels of moderators

Although we used the default options in forming the levels of the moderators, researchers can have a great deal of control on the levels formed in *manymome*. The function *mod_levels()* allows researchers to (a) use either *SD* (1 *SD* below mean, mean, and 1 *SD* above mean) or percentile (16th, 50th, and 84th percentiles) to form the levels, (b) specify distances in *SD* (values other than 1), (c) specify any numbers and values in *SD* for the levels, and (d) set levels using any values of the moderators when they have meaningful units (e.g., they are standard measures with commonly used cutoff values). For categorical moderators, researchers can set the group labels to other values. The output of *mod_levels()* for each moderator can then be combined by *merge_mod_levels()* into a single table and used as the value of *wlevels* in *cond_indirect_effects()* and proceed as in previous examples. Interested readers can refer to the help page of these functions and the examples at the OSF page for further information.

### Monte Carlo confidence intervals

Although nonparametric bootstrapping is the most popular method for forming the CI of an indirect effect or a conditional indirect effect, it is computationally intensive and requires the raw data. If raw data are not available or bootstrapping is too slow due to missing data or other issues, a viable alternative is the Monte Carlo method (Preacher & Selig, 2012). Instead of resampling the raw data to generate a large number of (bootstrap) estimates to form a CI, the estimated sampling variance–covariance matrix of the parameter estimates is used to simulate a large number of sets of sample parameter estimates, assuming multivariate normality.^15^ These simulated estimates are then used as in percentile bootstrap CI to form CI for an effect. When the sample size is large enough to have unbiased estimates of the sampling variances and covariances, and the sampling distribution of the estimates is close to multivariate normal, the Monte Carlo CI can also provide satisfactory performance (Pesigan & Cheung, 2020).^16^

All main functions presented above that support bootstrap CIs also support Monte Carlo CIs for models estimated by SEM.^17^ The only change is setting *mc_ci* to *TRUE* and using *mc_out* instead of *boot_out* to reuse generated estimates. Moreover, due to the much lower computational cost, it is common to set the number of replications to a much larger number. Therefore, *R* can be set to 10,000 or even 20,000. Because the workflow is virtually identical to that for bootstrap CI, we did not include an illustration here. Interested readers can refer to an illustration on the OSF page for this manuscript (https://osf.io/w9p87).

### Using multiple regression to estimate the parameters

**Fig. 5:**
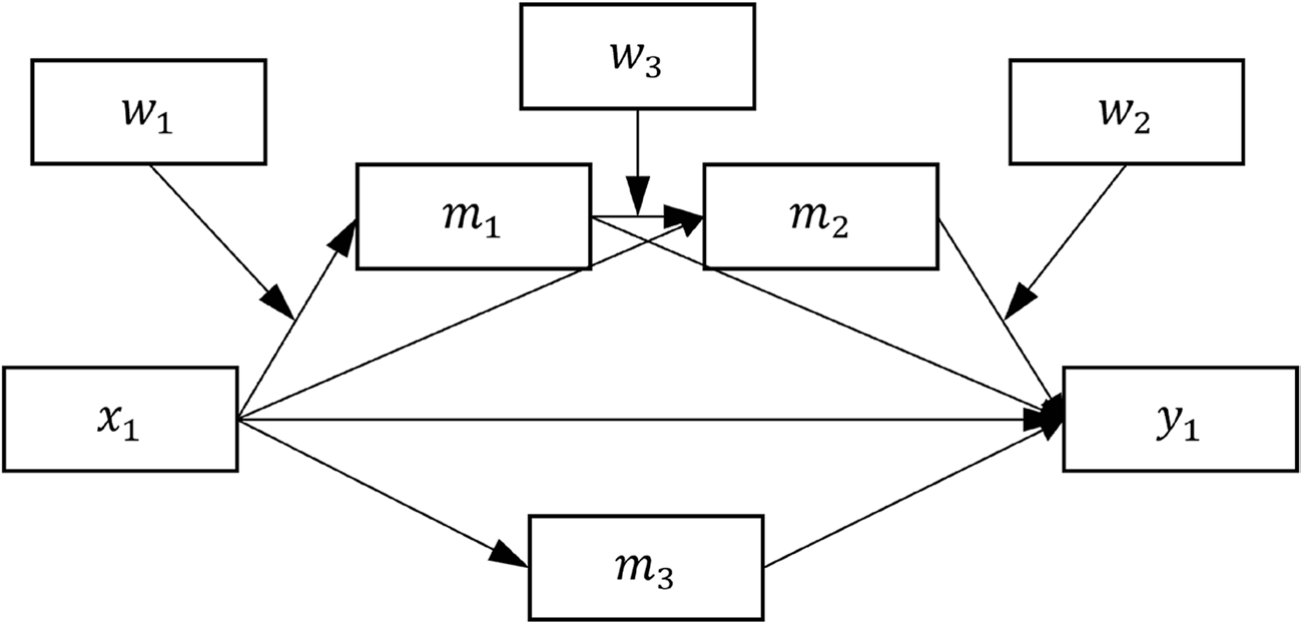
Model 3: The conceptual model (variances, covariances, and error terms omitted for readability)

Although we used SEM by *lavaan* to illustrate how *manymome* can be used on complicated models with missing data, if testing model fit is not a major concern and there are no missing data, researchers may prefer using multiple regression. This can be done in Step 1 by fitting all models by *lm()*. For example, suppose the model of interest is Model 3 (Fig. 5).

It has four endogenous variables (*m*_1_, *m*_2_, *m*_3_, and *y*_1_) and three moderators (*w*_1_, *w*_2_, and *w*_3_), and two control variables not shown (*c*_1_ and *c*_2_). The parameters can be estimated by the follow four regression models, assuming the data are stored in *dat*:lm_m1 <- lm(m1 ~ w1*x1 + c1 + c2, dat)lm_m2 <- lm(m2 ~ w3*m1 + x1 + c1 + c2, dat)lm_m3 <- lm(m3 ~ x1 + c1 + c2, dat)lm_y1 <- lm(y1 ~ w2*m2 + m1 + m3 + x1 + c1 + c2, dat)

The four outputs can be used as the value of the *fit* argument as in the previous examples by combining them as a list (e.g., *list(lm_m1, lm_m2, lm_m3, lm_y1)*). The functions *indirect_effect()* and *cond_indirect_effects()* will check whether the list of models (a) form a model with all variables connected to at least one other variable (i.e., they form a model of connected nodes) and (b) all analyses are based on the same sample—not just the same sample sizes but the same cases. All other steps, including bootstrapping, can be conducted as in the case of using *lavaan*. The use of *lm()* has the added benefit that, unlike *lavaan*, it natively supports categorical variables, and dummy variables are created automatically. Examples of using *lm()* with *manymome* can be found on the OSF page for this manuscript.

### Proportion of effect mediated

Alwin and Hauer (1975) proposed the proportion of effect mediated as a way to measure the indirect effect. For example, for a simple mediation with an *a*-path *x* → *m*, a *b*-path *m* → *y*, and a *c'*-path *x* → *y*, the total effect is *ab* + *c*^′^, the direct effect is *c*^′^, and the indirect effect is *ab*. The proportion of effect mediated is *ab*/(*ab* + *c*^′^). Miočević et al. (2018) showed that this measure is unstable (confidence interval coverage probabilities less than the nominal value) unless the sample size is large. Moreover, it is not interpretable when the effects from *x* to *y* do not have the same signs (some are positive, and some are negative). Nevertheless, when all the effects are of the same sign (all negative or all positive), this measure is easy to interpret and could be useful when used with other measures (e.g., standardized indirect effect). The function *indirect_proportion()* in *manymome* can be used to compute this proportion.^18^ It automatically computes the total effect from *x* to *y* along all pathways in a model and then computes the proportion of effect mediated along the requested pathway. It will also check the signs of all the effects and compute the proportion only if all the effects are of the same sign. Examples of using *indirect_proportion()* can be found on the OSF page for this manuscript (https://osf.io/2twx6).

## The strengths and limitations of *manymome* and other selected tools

Despite the flexibility and convenience of *manymome*, we do not think that any tool, including *manymome*, can replace all other tools. Therefore, we will highlight the strengths of *manymome* below in comparison to some existing tools, but also mention the limitations of the current version of *manymome*.

### The form of the model

We used a complicated model in Fig. 1 for illustration because, in practice, a model as complicated as or more complicated than that one is the norm rather than the exception. Some tools have limitations on the form of the model. For example, at the time of writing, although PROCESS can support a wide variety of models through the use of the *bmatrix*, *wmatrix*, and *zmatrix* arguments, using these arguments it can only support a model with at most six mediators (Hayes, 2022, p. 651). It supports as many as 10 mediators for some, but not all, numbered models, that is, the template models in PROCESS (Hayes, 2022, p. 588). For all models, numbered or user-defined, it only supports at most two moderators. Other tools have similar restrictions on the form of models. For regression-based mediation and moderation analysis, *manymome* has no limitation on these aspects of the models. Just fit all the regression models in a path model and then estimate and test any indirect effects and conditional indirect effects along any path. However, *manymome* does not yet officially support models in which a moderation effect is being moderated (e.g., Models 12 and 18 in PROCESS). For models readily supported by tools like PROCESS and *mediate()* in *mediation*, they are better tools, and the one-step approach can be sufficient.

If SEM is used to estimate model parameters in a model with mediation and moderation, SEM tools such as *lavaan* and *Amos* do not have the aforementioned limitations on the form of the models. To our knowledge, any valid models can be fitted, with few hard-coded limitations on the number of mediators, moderators, or paths. The *manymome* package can read the output of *lavaan* to compute the requested effects. Therefore, it also has very few inherent limitations on the number of predictors, mediators, outcome variables, moderators, or model form, except for models with moderating effects moderated by other moderators. Moreover, even if a model includes components that are not supported by *manymome*, it can still be used on paths that it supports, while other components are estimated and tested by other methods (e.g., defining user parameters).

### Types of variables

The types of variables supported also vary across tools. For example, PROCESS supports a wide variety of variables: predictors and moderators can be continuous or categorical, and outcome variables can be dichotomous. The *manymome* package does not officially support a path that starts or ends with a categorical variable when parameters are estimated by regression. If estimated by SEM using tetrachoric or polychoric correlations (by telling *lavaan* that some variables are ordered variables), *manymome* can be used when the predictors, mediators, and outcome variables are ordered categorical variables, because it works on the coefficients and does not need to know that they are based on tetrachoric or polychoric correlations. However, the results may not be as easy to interpret as those in regression-based analysis using tools like PROCESS. Therefore, when estimation is performed by SEM using *lavaan* with ordered categorical variables, the results of *manymome* are only as interpretable as the path coefficients of the component paths in the model. If in doubt, tools like PROCESS are better alternatives.

In the current version, *manymome* can be used for any mediator supported by *lavaan*. The *mediation* package by Tingley et al. (2014) supports categorical mediators by using appropriate regression models. PROCESS does not support categorical mediators at the time of writing (version 4.3). Nevertheless, if a mediator is set as *ordered* in *lavaan*, then *manymome* can compute the indirect effect through the latent variable hypothesized to underlie the categorical ordered variable. If researchers deem this interpretable, then *manymome* can be used. However, while SEM is flexible, and indirect effects through categorical mediators can be computed even in *lavaan* syntax, researchers need to decide whether the estimate of this indirect effect is interpretable in their datasets and models.

### Latent variables

For indirect effects, *manymome* fully supports models with latent variables. Indirect effects can be computed for any pathways with latent variables and/or observed variables. This is because functions such as *indirect_effect()* work by retrieving the corresponding path coefficients between two variables. If one or both variables are latent variables, they can still retrieve the correct coefficients. Confidence intervals can be formed as illustrated before. Because the only difference from the path analysis model examples is setting the arguments *x*, *y*, and *m* to the names of the latent variables, we did not include an illustration in the manuscript. Interested readers can refer to an illustration on the OSF project of this manuscript (https://osf.io/63adf).

For paths which involve latent variables and at least one moderator, *manymome* preliminarily supports some models. However, due to the complication in moderation that involves latent variables (for a review, see Cheung et al., 2021), more testing is needed before it is appropriate to introduce how to use *manymome* for moderation with latent variables. Researchers who want to use R to perform the analysis can use the *semTools* package (Jorgensen et al., 2022), which have functions for estimating and testing conditional effects among latent variables (see Schoemann & Jorgensen, 2021, for a detailed illustration). Another alternative is the factor score approach (Ng & Chan, 2020).

### Missing data

Missing data is a common phenomenon in behavioral research. However, some common tools cannot include cases with missing data and use listwise deletion, including only cases with complete data on all variables (e.g., PROCESS). The *manymome* package also has this limitation when the regression approach is used. It will check and require that exactly the same set of cases is used in all regression models. Listwise deletion will result in unnecessary loss in statistical power. If a model is fitted by SEM, then there are options to correctly handle missing data. As illustrated before, *lavaan* can use FIML to estimate parameters in the presence of missing data. Therefore, if SEM is used as the input of *manymome*, then *manymome* can also use all available cases in estimating the indirect and conditional indirect effects. For forming bootstrapping CI, *Amos* does not support bootstrapping in the presence of missing data (at the time of writing). There is no such limitation for *manymome* because *lavaan* supports combining bootstrapping with FIML. Therefore, by borrowing the strength of *lavaan*, *manymome* can form bootstrapping CIs for indirect and conditional indirect effects even in the presence of missing data.

Although FIML is a popular method to handle missing data in SEM, there are situations in which FIML may not have satisfactory performance, for example, when data are not multivariate normal (Enders, 2022). An alternative method, multiple imputation (Rubin, 1987), is a more flexible technique, though more difficult to use. Basically, it involves three steps. First, one or more methods are selected to impute values for missing data based on the posterior distribution of each variable with missing data, to generate *M* datasets with stochastically imputed values. Second, the complete-data method is then used to estimate the parameters in each imputed dataset. Lastly, results from the *M* datasets are pooled to form point estimates of the model parameters, as well as the estimated sampling variances and covariances of the pooled estimates.

It is beyond the scope of this manuscript to introduce multiple imputation. Interested readers are referred to van Buuren (2018) for a detailed introduction, and how to perform multiple imputation using the *mice* package. The *manymome* package supports models fitted in *lavaan* with multiple imputation, implemented by the *runMI()* function and its wrappers (e.g., *sem.mi()*) from the *semTools* package (Jorgensen et al., 2022).^19^ If requested, Monte Carlos CIs can also be formed using the sampling variance and covariance matrix computed from the output of *runMI()*. The process is done internally and automatically. Users simply use the output of *runMI()* or *sem.mi()* in place of the output of *lavaan::sem()*. If the function of *manymome* detects that the fit object is of the class *lavaan.mi*, it will use the appropriate functions to extract the pooled point estimates to compute the requested effects and extract the pooled estimates of the sampling variances and covariances of the parameter estimates to form Monte Carlo CIs. A numerical example can be found at the OSF page of this manuscript (https://osf.io/85gpj).

### Standardized effects

The support for standardized indirect effects and conditional indirect effects varies across tools. The *mediate()* function of *mediation* does not support standardized effects. PROCESS can report standardized indirect effects and correct bootstrap CIs that take into account the sampling variation of the *SD*s of the variables (add *stand = 1*). However, it does not support standardized conditional indirect effects for models with moderators (Hayes, 2022, p. 598). The *manymome* package forms the bootstrap CIs of standardized effects correctly because it uses the *SD*s in each bootstrap samples to perform the standardization and supports standardized effects in mediation models, moderation models, and moderated mediation models.

The case for SEM tools such as *lavaan* and *Amos* is more complicated. For standardized indirect effects, although *lavaan* can report the standardized estimates through *standardizedSolution()*, the confidence interval for the standardized indirect effect is actually a symmetric one based on the delta method (the same method used to derive the Sobel test, which is now usually not recommended for indirect effects because it assumes the sampling distribution to be symmetric, which is only asymptotically true), even if the bootstrapping confidence interval is requested by *se = "boot"* (at the time of writing, in version 0.6-15). They are not the percentile bootstrap CIs as reported in tools like PROCESS for indirect effects. On the other hand, *Amos* can report correct bootstrap CIs for standardized indirect effects. The package *manymome* forms the CI itself even when *lavaan* is used, and so the bootstrap CIs are always the percentile CIs as requested.

For standardized conditional indirect effects, in principle, they can be estimated in both *lavaan* and *Amos*. However, in practice, both tools require users to define the estimate manually as described above. The package *manymome* uses the model-implied *SD*s reported by *lavaan* in each bootstrap sample and so the computation is simpler, more reliable, and automatic. It also reports the computation formulas so that researchers can verify the computation themselves if necessary.

Lastly, we would like to note that standardized conditional effects (direct or indirect) are different from the standardized *coefficient* of a product term. The *manymome* package can only compute the former and form its confidence interval. The latter is the coefficient of a product term on the standardized metric, called the *standardized moderation effect* (Cheung et al., 2022), and *manymome* cannot be used to compute this (for now). In OLS regression, the proper way to form the standardized moderation effect is to standardize the variables before forming the product term (Aiken & West, 1991; Friedrich, 1982). The case is the same in SEM. However, this properly standardized coefficient of the product term can also be computed from the standard deviations of the moderator, the predictor, and the outcome variable (see Cheung et al., 2022, for the derivation). The package *stdmod* presented in Cheung et al. was developed for this purpose. It can compute the standardized moderation effect in both OLS regression models fitted by *lm()* and models fitted by *lavaan*, and form nonparametric bootstrap CIs for the coefficient, which is not normally distributed because its computation involves three sample standard deviations, just like the case for standardized regression coefficients (Yuan & Chan, 2011). Researchers interested in the standardized coefficient of the product term can refer to Cheung et al. (2022) and the package *stdmod*.

### The pedagogical risk of automation

The goal of *manymome*, like tools such as PROCESS, is to automate tasks that can be done manually using syntax. However, pedagogically, automation can make researchers and students less motivated to learn the computation process. For example, the computation of conditional effects and conditional indirect effects can be done manually. Therefore, we would like to recommend that researchers learn how to compute the effects first (e.g., Aiken & West, 1991; Hayes, 2022) before using tools that automate the tasks.

To encourage users to understand (and verify) the computation, the printout of *indirect_effect()* by default shows how the computation is done. The printout of the output of *cond_indirect_effects()* by default only shows the component effects for readability. However, the lower-level function *cond_indirect()*, used by *cond_indirect_effects()*, also prints the computation of a conditional effect or conditional indirect effect. For example, researchers can use *get_one_cond_indirect_effect()* to show the details of a specific conditional indirect effect.> get_one_cond_indirect_effect(out_cond, 1)== Conditional Indirect Effect == Path: x1 -> m1 -> m2 -> y2 Moderators: w1 Conditional Indirect Effect: -0.009 95.0% Bootstrap CI: [-0.041 to 0.022] When: w1 = 1.011Computation Formula: (b.m1~x1 + (b.w1x1)*(w1))*(b.m2~m1)*(b.y2~m2)Computation: ((0.35235) + (0.15057)*(1.01052))*(0.54836)*(-0.03304)Percentile confidence interval formed by nonparametric bootstrapping with 5000 bootstrap samples.Coefficients of Component Paths: Path Conditional Effect Original Coefficient m1~x1 0.505 0.352 m2~m1 0.548 0.548 y2~m2 -0.033 -0.033

The first argument is the output of *cond_indirect_effects()*, and the second argument is the row number of the conditional effect to be extracted. Similar to *indirect_effect()*, it shows how the conditional effect is computed. We made the computation displayed by default because we encourage researchers to examine how an effect is computed.

## Conclusion

Each tool has its own advantages and disadvantages. Our goal in developing *manymome* is to provide an alternative tool that uses a two-step workflow, separating the parameter estimation step from the indirect and conditional indirect effect steps. This gives researchers more freedom in the models to be fitted, and reduces the need to define parameters in advance, which can be complicated, error-prone, and time-consuming, especially when bootstrapping is used with missing data and standardized effects are needed. Nevertheless, there are cases in which other tools are more appropriate and the one-step workflow is more convenient, or the models and variables are supported by other tools but not yet supported by *manymome*. This is why we started the name with *many*: It does support many models, but it does not support *all* models (very few tools can). The two-step workflow has the advantage that, rather than developing a tool that performs both parameter estimation and the computation of indirect and conditional indirect effects, it leverages the power of existing tools such as *lavaan* in parameter estimation and model fitting and focuses on how to use these estimates to compute and test the indirect and conditional indirect effects. Further development of *manymome* can extend its support to more types of variables and models by focusing on how to compute the requested effects from the parameter estimates of other tools, without the need to reinvent tools to perform the parameter estimation for these models and variables. Development is also underway to add support for sensitivity analysis (Pek & MacCallum, 2011), to identify cases which are influential on the estimates of indirect effects or conditional indirect effects.^20^

## Footnotes

1 It is because scale change in mediators will be canceled out in the multiplication.

2 The indirect effect with both the predictor and the outcome variable standardized is also called *completely standardized indirect effect*. If there is no ambiguity, we simply call this effect *standardized indirect effect*.

3 The standardized solution of most SEM programs, such as *lavaan*, cannot be used for computing standardized conditional indirect effects because they standardize the product term, not its components.

4 We need to stress that our goal in adopting this two-stage approach is not to encourage hypothesizing after the results are known (HARKing, see Kerr, 1998). We believe preregistration should be encouraged, and data-driven exploration is acceptable as long as it is reported as such. One of the goals for the two-stage approach is to address the technical issues mentioned. Researchers should not use the package for cherry-picking. (We would like to thank an anonymous reviewer for raising this concern on how the package may be misused.)

5 We do not use the colon (:) operator because covariances between the product terms and other variables need to be added manually as Kwan and Chan (2018) did. This cannot be done for terms created by the colon operator.

6 We used *R* to name this argument because this is the name of the same argument in *boot()*, the function that came with R for bootstrapping.

7 Note that the results are reproducible when parallel processing is used only if the configuration of the bootstrapping does not change, for example, has the same number of processes and ran on the same OS.

8 The function *get_prod()* was used internally to identify product term(s) in a dataset, by checking each variable/column in the data to see whether it was the product of other variables/columns. The algorithm used is described in a technical appendix available at the OSF project for this manuscript: https://osf.io/9xys5 for the PDF version viewable online, and https://osf.io/hkxwv for the HTML version that needed to be downloaded first but is more readable.

9 Although an indirect effect is commonly tested using a confidence interval, some researchers may also want to report a *p*-value. This can be done by calling *print()* directly to print the output, setting the argument *pvalue* to *TRUE*. The approach by Asparouhov and Muthén (2021) is used to compute a bootstrap *p*-value for each effect. This value is *not* the *p*-value under the null hypothesis. Instead, it is the value at which the (1 – *p*)% bootstrap confidence will have one of its limits equal to (or nearly equal to) zero. We thank an anonymous reviewer for suggesting this feature.

10 Note that *w*_2_, though a moderator in the model, is not involved in the path from *x*_1_ → *m*_1_ → *m*_2_ → *y*_2_. Therefore, its value has no impact on the conditional indirect effect along this path.

11 The package *semTools* (Jorgensen et al., 2022) also supports probing interaction through the *lavaan2emmeans()* function, developed by Ben-Shachar (2022), through the *emmeans* package (Lenth, 2023).

12 Other estimation can also be used based on the indicator variables used. However, this has little impact on the illustration because the computation and the bootstrap CIs only rely on the point estimation, not on the standard errors.

13 User-supplied group names can be used, presented later. For example, users can change the default name for the reference group "Reference," back to "Alpha."

14 The operation will check whether the bootstrap estimates in the effects being combined are exactly the same, to ensure that the bootstrap CI is based on the same set of bootstrap samples.

15 Note that it is the distribution of the *sample estimates* which is assumed to be multivariate normal, not the population distribution of the variables. This assumption is made when forming the default CIs of parameter estimates in commonly used estimation methods of SEM, including robust methods such as sandwich CIs for nonnormal distributed variables.

16 For standardized indirect effects, the standard deviation of the outcome variable is needed. It is not a parameter and so cannot be directly simulated. When forming Monte Carlo CI for standardized indirect effects, the simulated parameter estimates are used to compute the implied standard deviation of the outcome variable. This method has been shown to have satisfactory performance for standardized indirect effects (Pesigan & Cheung, 2023).

17 We thank an anonymous reviewer for suggesting this feature for the package.

18 We thank an anonymous reviewer for suggesting this feature.

19 We thank an anonymous reviewer for suggesting this feature.

20 We would like to thank an anonymous reviewer for making this suggestion.

## References

[CR1] Aiken, L. S., & West, S. G. (1991). *Multiple regression: Testing and interpreting interactions*. SAGE Publication.

[CR2] Alwin, D. F., & Hauser, R. M. (1975). The decomposition of effects in path analysis. *American Sociological Review, 40*(1), 37. 10.2307/2094445 10.2307/2094445

[CR3] Amos Development Corporation. (2021). *User-defined estimands*. Retrieved from http://amosdevelopment.com/features/user-defined/index.html on August 8, 2022.

[CR4] Asparouhov, A., & Muthén, B. (2021). Bootstrap *p*-value computation. Retrieved March 8, 2023, from https://www.statmodel.com/download/FAQ-Bootstrap%20-%20Pvalue.pdf

[CR5] Arbuckle, J. L. (1996). Full information estimation in the presence of incomplete data. In G. A. Marcoulides & R. E. Schumacker (Eds.), *Advanced structural equation modeling: Issues and techniques* (pp. 243–277). Lawrence Erlbaum Associates.

[CR6] Arbuckle, J. L. (2021). *IBM® SPSS® Amos™ 28 user’s guide*.

[CR7] Baron, R. M., & Kenny, D. A. (1986). The moderator-mediator variable distinction in social psychological research: Conceptual, strategic, and statistical considerations. *Journal of Personality and Social Psychology, 51*(6), 1173–1182. 3806354 10.1037/0022-3514.51.6.1173

[CR8] Ben-Shachar, M. S. (2022). *lavaan2emmeans*. A function in the *semTools* package (T. D. Jorgensen, S. Pornprasertmanit, A. M. Schoemann, & Y. Rosseel), version 0.5-6.

[CR9] Chamorro-Premuzic, T., & Furnham, A. (2008). Personality, intelligence and approaches to learning as predictors of academic performance. *Personality and Individual Differences, 44*(7), 1596–1603. 10.1016/j.paid.2008.01.003 10.1016/j.paid.2008.01.003

[CR10] Cheung, G. W., Cooper-Thomas, H. D., Lau, R. S., & Wang, L. C. (2021). Testing moderation in business and psychological studies with latent moderated structural equations. *Journal of Business and Psychology, 36*(6), 1009–1033. 10.1007/s10869-020-09717-0 10.1007/s10869-020-09717-0

[CR11] Cheung, M. W.-L. (2009). Comparison of methods for constructing confidence intervals of standardized indirect effects. *Behavior Research Methods, 41*(2), 425–438. 10.3758/BRM.41.2.425 19363183 10.3758/BRM.41.2.425

[CR12] Cheung, S. F., Cheung, S.-H., Lau, E. Y. Y., Hui, C. H., & Vong, W. N. (2022). Improving an old way to measure moderation effect in standardized units. *Health Psychology, 41*(7), 502–505. 10.1037/hea0001188 35467898 10.1037/hea0001188

[CR13] Craig, C. C. (1936). On the frequency function of *xy*. *The Annals of Mathematical Statistics, 7*(1), 1–15. 10.1214/aoms/1177732541 10.1214/aoms/1177732541

[CR14] Efron, B., & Tibshirani, R. (1993). *An introduction to the bootstrap*. Chapman & Hall/CRC.

[CR15] Enders, C. K. (2022). *Applied missing data analysis* (2^nd^ ed.). The Guilford Press.

[CR16] Falk, C. F. (2018). Are robust standard errors the best approach for interval estimation with nonnormal data in structural equation modeling? *Structural Equation Modeling: A Multidisciplinary Journal, 25*(2), 244–266. 10.1080/10705511.2017.1367254 10.1080/10705511.2017.1367254

[CR17] Friedrich, R. J. (1982). In defense of multiplicative terms in multiple regression equations. *American Journal of Political Science, 26*(4), 797–833. 10.2307/2110973

[CR18] Hayes, A. F. (2015). An index and test of linear moderated mediation. *Multivariate Behavioral Research, 50*(1), 1–22. 10.1080/00273171.2014.962683 26609740 10.1080/00273171.2014.962683

[CR19] Hayes, A. F. (2018). Partial, conditional, and moderated moderated mediation: Quantification, inference, and interpretation. *Communication Monographs, 85*(1), 4–40. 10.1080/03637751.2017.1352100 10.1080/03637751.2017.1352100

[CR20] Hayes, A. F. (2022). *Introduction to mediation, moderation, and conditional process analysis: A regression-based approach* (3^rd^ ed.). The Guilford Press.

[CR21] Jorgensen, T. D., Pornprasertmanit, S., Schoemann, A. M., & Rosseel, Y. (2022). *semTools*: Useful tools for structural equation modeling. R package version 0.5-6. Retrieved May 13, 2022, from https://CRAN.R-project.org/package=semTools

[CR22] Kerr, N. L. (1998). HARKing: Hypothesizing after the results are known. *Personality and Social Psychology Review, 2*(3), 196–217. 10.1207/s15327957pspr0203_4 15647155 10.1207/s15327957pspr0203_4

[CR23] Kline, R. B. (2023). *Principles and practice of structural equation modeling* (5th ed.). The Guilford Press.

[CR24] Kwan, J. L. Y., & Chan, W. (2018). Variable system: An alternative approach for the analysis of mediated moderation. *Psychological Methods, 23*(2), 262–277. 10.1037/met0000160 29172615 10.1037/met0000160

[CR25] Lenth, R. V. (2023). *emmeans*: Estimated marginal means, aka least-squares means. R package version, 1.8.5. Retrieved March 8, 2023, from https://CRAN.R-project.org/package=emmeans

[CR26] Miles, J. N. V., Kulesza, M., Ewing, B., Shih, R. A., Tucker, J. S., & D’Amico, E. J. (2015). Moderated mediation analysis: An illustration using the association of gender with delinquency and mental health. *Journal of Criminal Psychology, 5*(2), 99–123. 10.1108/JCP-02-2015-0010 26500722 10.1108/JCP-02-2015-0010 PMC4616155

[CR27] Miočević, M., O’Rourke, H. P., MacKinnon, D. P., & Brown, H. C. (2018). Statistical properties of four effect-size measures for mediation models. *Behavior Research Methods, 50*(1), 285–301. 10.3758/s13428-017-0870-1 28342072 10.3758/s13428-017-0870-1 PMC5809552

[CR28] Muthén, L. K., & Muthén, B. O. (2017). *Mplus 8 user’s guide*.

[CR29] Ng, J. C. K., & Chan, W. (2020). Latent moderation analysis: A factor score approach. *Structural Equation Modeling: A Multidisciplinary Journal, 27*(4), 629–648. 10.1080/10705511.2019.1664304 10.1080/10705511.2019.1664304

[CR30] Pek, J., & MacCallum, R. (2011). Sensitivity analysis in structural equation models: Cases and their influence. *Multivariate Behavioral Research, 46*(2), 202–228. 10.1080/00273171.2011.561068 26741328 10.1080/00273171.2011.561068

[CR31] Pesigan, I. J. A., & Cheung, S. F. (2020). SEM-based methods to form confidence intervals for indirect effect: Still applicable given nonnormality, under certain conditions. *Frontiers in Psychology, 11*. 10.3389/fpsyg.2020.571928 10.3389/fpsyg.2020.571928 PMC7775588 33391086

[CR32] Pesigan, I. J. A., & Cheung, S. F. (2023). Monte Carlo confidence intervals for the indirect effect with missing data. *Behavior Research Methods*. 10.3758/s13428-023-02114-4 10.3758/s13428-023-02114-4 37550469

[CR33] Preacher, K. J., Rucker, D. D., & Hayes, A. F. (2007). Addressing moderated mediation hypotheses: Theory, methods, and prescriptions. *Multivariate Behavioral Research, 42*(1), 185–227. 10.1080/00273170701341316 26821081 10.1080/00273170701341316

[CR34] Preacher, K. J., & Selig, J. P. (2012). Advantages of Monte Carlo confidence intervals for indirect effects. *Communication Methods and Measures, 6*(2), 77–98. 10.1080/19312458.2012.679848 10.1080/19312458.2012.679848

[CR35] R Core Team. (2022). *R: A language and environment for statistical computing*. R Foundation for Statistical Computing https://www.R-project.org/

[CR36] Rosseel, Y. (2012). Lavaan: An R package for structural equation modeling. *Journal of Statistical Software, 48*(2). Retrieved April 12, 2013, from http://www.jstatsoft.org/v48/i02/paper

[CR37] Rubin, D. B. (1987). *Multiple imputation for nonresponse in surveys*. Wiley.

[CR38] Scott, H., & Woods, H. C. (2018). Fear of missing out and sleep: Cognitive behavioural factors in adolescents’ nighttime social media use. *Journal of Adolescence, 68*(1), 61–65. 10.1016/j.adolescence.2018.07.009 30031979 10.1016/j.adolescence.2018.07.009

[CR39] Schoemann, A. M., & Jorgensen, T. D. (2021). Testing and interpreting latent variable interactions using the *semTools* package. *Psych, 3*(*3*), *Article 3*. 10.3390/psych3030024 10.3390/psych3030024

[CR40] Sobel, M. E. (1982). Asymptotic confidence intervals for indirect effects in structural equation models. *Sociological Methodology, 13*, 290. 10.2307/270723 10.2307/270723

[CR41] Tingley, D., Yamamoto, T., Hirose, K., Keele, L., & Imai, K. (2014). Mediation: R package for causal mediation analysis. *Journal of Statistical Software, 59*(5), 1–38. 10.18637/jss.v059.i0526917999 10.18637/jss.v059.i05

[CR42] Van Buuren, S. (2018). *Flexible imputation of missing data* (2nd ed.). Taylor and Francis Group: CRC Press.

[CR43] Yuan, K.-H., & Chan, W. (2011). Biases and standard errors of standardized regression coefficients. *Psychometrika, 76*(4), 670–690. 10.1007/s11336-011-9224-6 27519686 10.1007/s11336-011-9224-6

